# Transcriptome of the floral transition in *Rosa chinensis* ‘Old Blush’

**DOI:** 10.1186/s12864-017-3584-y

**Published:** 2017-02-23

**Authors:** Xuelian Guo, Chao Yu, Le Luo, Huihua Wan, Ni Zhen, Tingliang Xu, Jiongrui Tan, Huitang Pan, Qixiang Zhang

**Affiliations:** Beijing Key Laboratory of Ornamental Plants Germplasm Innovation & Molecular Breeding, National Engineering Research Center for Floriculture, Beijing Laboratory of Urban and Rural Ecological Environment, Key Laboratory of Genetics and Breeding in Forest Trees and Ornamental Plants of Ministry of Education, School of Landscape Architecture, Beijing Forestry University, Beijing, 100083 China

**Keywords:** Floral transition, Circadian clock, Sugar signaling, Hormone signaling, Recurrent flowering, Differentially expressed genes

## Abstract

**Background:**

The floral transition plays a vital role in the life of ornamental plants. Despite progress in model plants, the molecular mechanisms of flowering regulation remain unknown in perennial plants. *Rosa chinensis* ‘Old Blush’ is a unique plant that can flower continuously year-round. In this study, gene expression profiles associated with the flowering transition were comprehensively analyzed during floral transition in the rose.

**Results:**

According to the transcriptomic profiles, 85,663 unigenes and 1,637 differentially expressed genes (DEGs) were identified, among which 32 unigenes were involved in the circadian clock, sugar metabolism, hormone, and autonomous pathways. A hypothetical model for the regulation of floral transition was proposed in which the candidate genes function synergistically the floral transition process. Hormone contents and biosynthesis and metabolism genes fluctuated during the rose floral transition process. Gibberellins (GAs) inhibited rose floral transition, the content of GAs gradually decreased and *GA2ox* and *SCL13* were upregulated from vegetative (VM) meristem to floral meristem (FM). Auxin plays an affirmative part in mediating floral transition, auxin content and auxin-related gene expression levels were gradually upregulated during the floral transition of the rose. However, ABA content and ABA signal genes were gradually downregulated, suggesting that ABA passively regulates the rose floral transition by participating in sugar signaling. Furthermore, sugar content and sugar metabolism genes increased during floral transition in the rose, which may be a further florigenic signal that activates floral transition. Additionally, *FRI*, *FY*, *DRM1*, *ELIP*, *COP1*, *CO*, and *COL16* are involved in the circadian clock and autonomous pathway, respectively, and they play a positively activating role in regulating floral transition. Overall, physiological changes associated with genes involved in the circadian clock or autonomous pathway collectively regulated the rose floral transition.

**Conclusions:**

Our results summarize a valuable collective of gene expression profiles characterizing the rose floral transition. The DEGs are candidates for functional analyses of genes affecting the floral transition in the rose, which is a precious resource that reveals the molecular mechanism of mediating floral transition in other perennial plants.

**Electronic supplementary material:**

The online version of this article (doi:10.1186/s12864-017-3584-y) contains supplementary material, which is available to authorized users.

## Background

Roses have been used as garden ornamental plants and cut flowers for centuries, which are characterized by recurrent flowering; however, little is known about the genetic and molecular basis of the floral transition in the species. The timing of the floral transition is mediated by complex regulatory networks that constantly monitor environmental and endogenous cues.

Enormous progress has been made in research on the genetic, epigenetic and environmental factors that trigger the transition from vegetative growth to flowering in the model plant *Arabidopsis thaliana*. Environmental factors, photoperiod, and vernalization pathways mediate the transition to flowering in cooperation with diverse exogenous cues, including autonomous, gibberellin (GA), trehalose-6-phosphate (T6P), and age-dependent pathways [1, 2]. In combination, all these pathways converge to mediate a set of “floral integrator genes,” including *FLOWERING LOCUS T* (*FT*), *SUPPRESSOR OF OVEREXPRESSION OF CONSTANS1 (SOC1), CONSTANS (CO), FLOWERING LOCUS C (FLC)*, and the meristem identity genes *LEAFY (LFY)*, *APETALA1 (AP1)*, and *FRUITFULL (FUL)*, which irreversibly confer the transition from the vegetative to the reproductive meristem [2, 3]. However, perennial plants do not die after flowering; instead, they appropriately accomplish the conversion from vegetative to reproductive development once or multiple times per year. Studies of annual plants cannot completely uncover the mechanisms of floral transition that underlie perennial plants, such as the rose recurrent flowering.

A few flowering genes have recently been identified in the rose. EST sequencing using cDNA libraries has been used to identify candidate genes, e.g., *RoCOL*, *RoRGA*, *RoGI*, and *RoSOC1*, involved in rose floral transition [4]. Plant hormone signal transduction is involved in the floral transition process in *A. thaliana* [5, 6], and auxin, ethylene, and gibberellin signaling genes are also involved in rose floral transition [5, 7]. In contrast, the role of GA in flowering in perennial plants is inconsistent with its role in *Arabidopsis* [8, 9]. GA is an inhibitor of floral transition in nonrecurrent roses, GA metabolism genes, *Ro*GA20ox, encoding an enzyme of active GA synthesis, was down-regulated in floral transition, whereas *Ro*GA2ox, encoding a GA inactivation enzyme, was upregulated [9]. The *TFL1* homology *RoKSN*, was reported to regulate continuous flowering in the rose, and the function of *RoKSN* caused continuous flowering [10]. The application of GA_3_ promoted the accumulation of *RoKSN* in nonrecurrent roses during spring, while it inhibited floral transition. However, it had no function during summer, while other factors control *RoKSN* in nonrecurrent rose. In the recurrent rose, due to the insertion of a *copia* retrotransposon, the expression level of *RoKSN* was kept low year-round, and exogenous GA_3_ did not affect the floral transition in recurrent rose at any time [7]. Randoux, et al. [11] validated that ectopic expression of *RoKSN* impeded the floral induction in *R. hybrid* RI. However, the *KSN* ^*copia*^ allele has not been found in *R. rugosa* ‘Hamanasu’, which can also flower continuously. This suggests that *RoKSN* is not the only factor that controls the trait of recurrent flowering [12]; it is likely that other factors can affect the character. *Rosa chinensis* ‘Old Blush’ is a common ancestor of modern roses, and exhibits recurrent flowering, and may thus provide the best material to study the molecular mechanism of floral transition in the rose.

The roles of several key regulatory genes involved in the rose floral transition have been examined; however, the composition and mechanisms of the underlying global regulatory networks at the transcriptome level are still poorly understood. We used a high-throughput next-generation sequencing platform to sequence cDNA libraries at three stages of the rose flower transition process. We mined global differentially expressed genes (DEGs) or novel transcripts and isoforms involved in the rose floral transition. Our results demonstrated that the DEGs between the VM and TM stages play a key role in regulating floral transition. These results provide a comprehensive understanding of the molecular mechanisms that mediate the floral transition in rose.

## Results

### Morphological description of the rose flowering transition

**Fig. 1:**
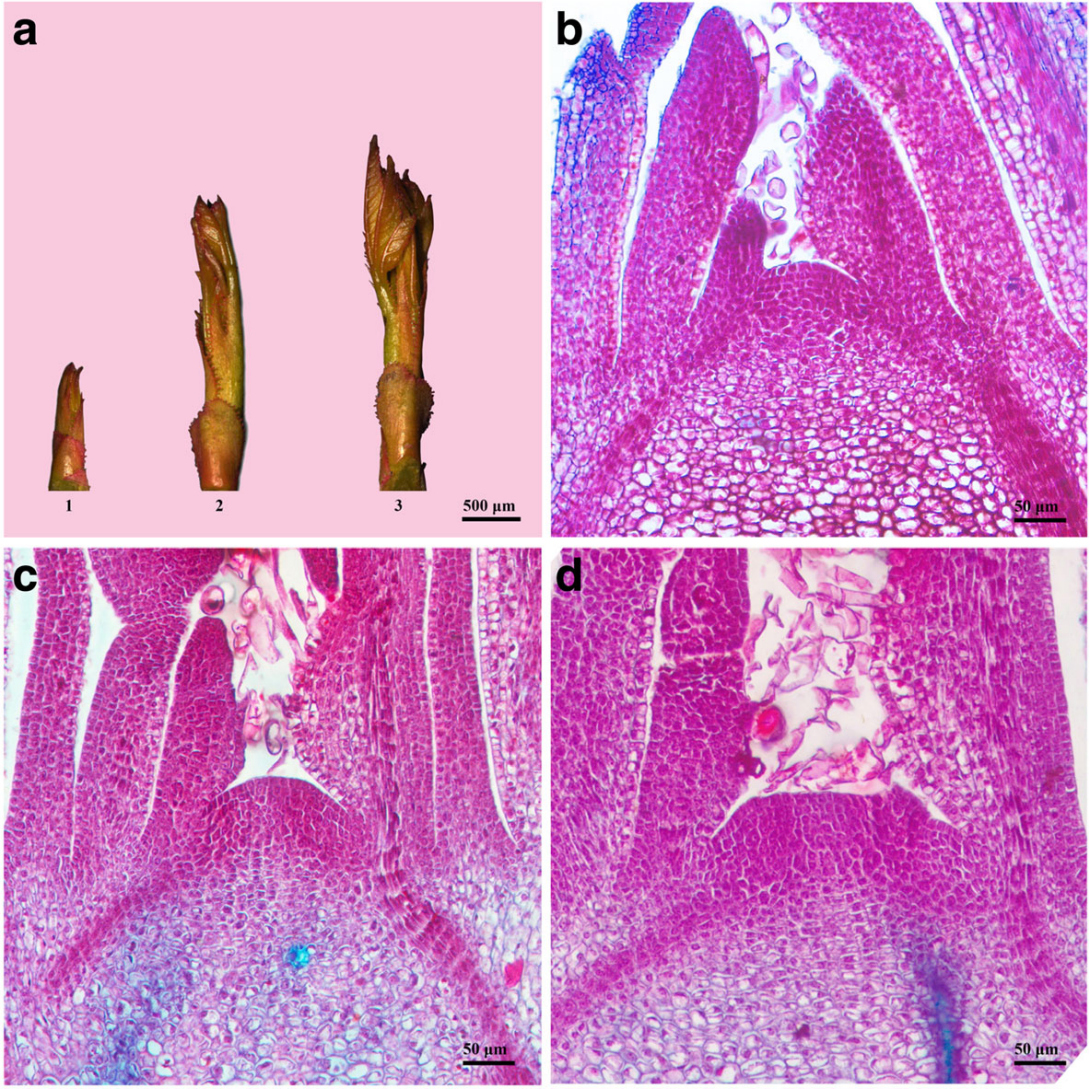
Morphology change of floral transition in rose. **a** Shown are axillary buds from *Rosa chinensis* ‘Old Blush’ at different stages. The floral transition process was analyzed at a histological level: vegetative meristem, VM (**b**); pre-floral meristem, TM (**c**); floral meristem, FM (**d**)

**Fig. 2:**
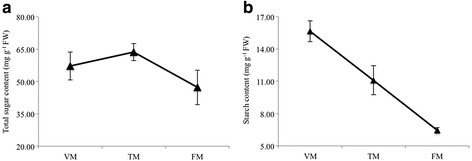
Total sugar and starch contents of shoots during the floral transition process in the rose. **a** Sugar content and **b** starch content. Values are means of three replicates ± SE

**Fig. 3:**
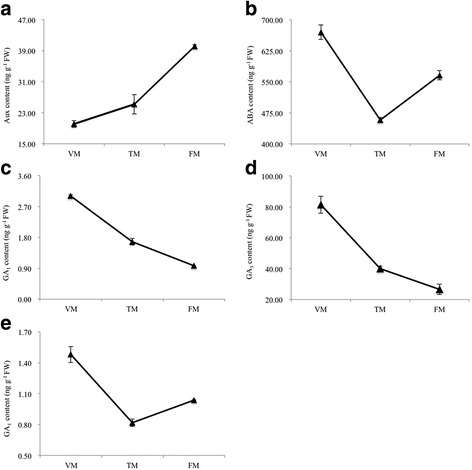
Hormone content of shoots during the floral transition process in rose. **a** Auxin (Aux); **b** Abscisic acid (ABA); **c** Gibberellin acid 1 (GA_1_); **d** Gibberellin acid 3 (GA_3_); **e** Gibberellin acid 4 (GA_4_)

Based on the morphological changes in the shoot apical meristem (SAM), we divided the continuous differentiation process from the vegetative to reproductive meristem into three stages in *R. chinensis* ‘Old Blush’ as follows: vegetative meristem (VM), pre-floral meristem (TM), and floral meristem (FM) (Fig. 1 and Additional file 1). Initially, at the VM stage, the shoot length was less than or equal to 0.5 cm, and meristems were flat and narrow (Fig. 1a-1 and b). At TM, meristems became broader and hunched into a dome shape, with shoots of 1.0–1.1 cm; the first 5-leaflet leaf was visible, but did not unfold (Fig. 1a-2 and c). At conic apices, the primordia were positioned higher than those at the VM stage. This was designated the floral transition stage, at which the shoot apex transformed from vegetative to reproductive growth. At the FM stage, the first 5-leaflet leaf prepared to unfold and the shoots were longer than 1.5 cm (Fig. 1a-3). Importantly, the sepal primordia were visible (Fig. 1d), the meristem initiated flower development, and differentiated sepals, petals, pistils, and so on were observed (Additional file 1).

### Sugar and hormone contents during the flower transition process

The total sugar and starch levels were analyzed in the shoots at three stages during the flowering transition process. In shoots, the total sugar content increased by 11.2% from VM to TM, but decreased by 25.7% between TM and FM (Fig. 2a). In addition, the starch content decreased by 29.1% between VM and TM, and then decreased sharply by 41.9% from TM to FM (Fig. 2b).

The levels of hormones were also measured in shoots at three points during the flowering transition process (Fig. 3). The Auxin (Aux) content increased by 25.2% between VM and TM, and increased by an additional 59.3% from TM to FM (Fig. 3a). The ABA content decreased by 31.7% between TM and FM, and increased by 23.6% from TM to FM (Fig. 3b). GA_1_ and GA_3_ contents exhibited a similar trend, decreasing sharply by 44.5% and 50.8%, respectively, from VM to TM, and decreasing by 41.9 and 33.6%, respectively, between TM and FM (Fig. 3c and d). GA_4_ decreased by 44.6% from VM and TM, but increased by 26.8% between VM and TM (Fig. 3e).

### Sequencing, assembly, and annotation of the rose transcriptome

**Fig. 4:**
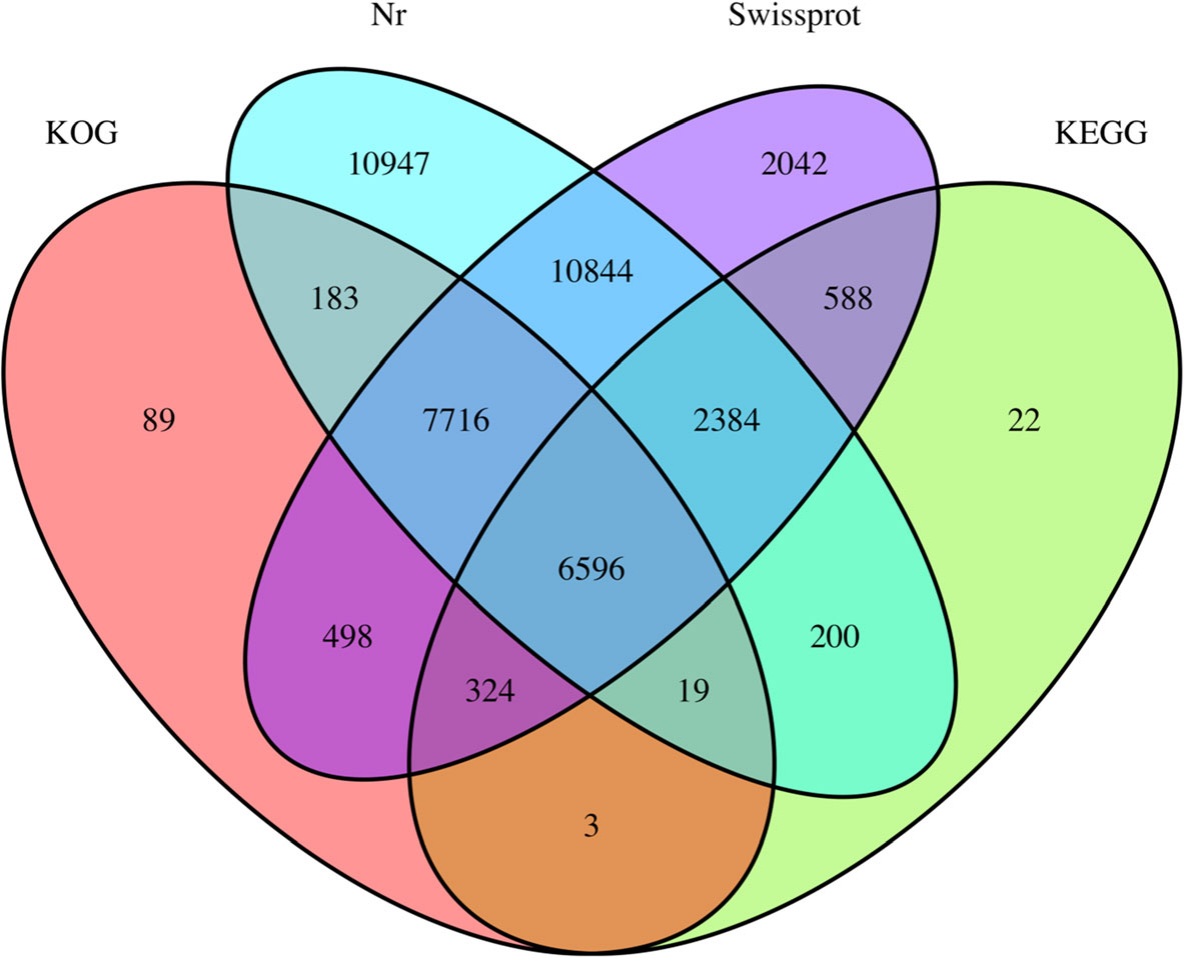
Veen diagram of number of unigenes annotated by BLAXTx against protein databases

A total of 568,805,892 raw reads were obtained. After low-quality reads were filtered out, 550,108,308 clean reads were selected for further analysis (Table 1). Finally, 85,663 unigenes with a mean size of 814 bp were assembled, which lengths ranging from 201 to 17,109 bp (Additional file 2). In total, 57.98% of the unigenes were annotated using at least one database with an E-value threshold of <0.5 (Additional file 3A and B); the database annotation results are summarized in Fig. 4. Among 85,663 unigenes, 38,884 (45.39%) and 30,992 (36.17%) were annotated using the NCBI Nr database and the Swiss-Prot protein database, respectively. Based on a GO analysis, 28,794 (33.61%) unigenes were successfully annotated using gene ontology (GO) assignments and classified into three GO categories: cellular component, biological process, and molecular function (Additional file 4). In addition, 15,309 (17.87%) unigenes were assigned to 279 pathways using the Kyoto Encyclopedia of Genes and Genomes (KEGG) database (Additional file 5).

**Table 1:** Throughput and quality of RNA-seq of DGE libraries

Library	Raw reads	Clean reads	Q20 (%)	Q30 (%)	GC content (%)
VM_YYF1	65602696	63238540	95.95	90.46	46.68
VM_YYF2	57636726	55841616	95.72	89.93	46.74
VM_YYF3	63510658	61791438	95.71	89.87	46.94
TM_YYF1	55705676	53913152	95.69	89.74	46.72
TM_YYF2	74110108	71678314	95.47	89.28	46.64
TM_YYF3	62125606	60033978	95.77	89.84	46.82
FM_YYF1	69117430	65960198	95.61	89.61	46.58
FM_YYF2	56955366	55430114	94.67	87.65	46.65
FM_YYF3	64041626	62220958	95.38	89.08	46.87

### Identification of differentially expressed genes using digital gene expression tags

The repeatability of the differential gene expression (DGE) libraries was evaluated using a PCA analysis. The TM_YYF1 and FM_YYF2 libraries did not form a cluster with two other replicates (Additional file 6A). To improve the repeatability between replicates at the three stages, the TM_YYF1 and FM_YYF2 libraries were eliminated and the PCA analysis was repeated. This improved the repeatability between replicates and discrepancy between groups (Additional file 6B).

**Fig. 5:**
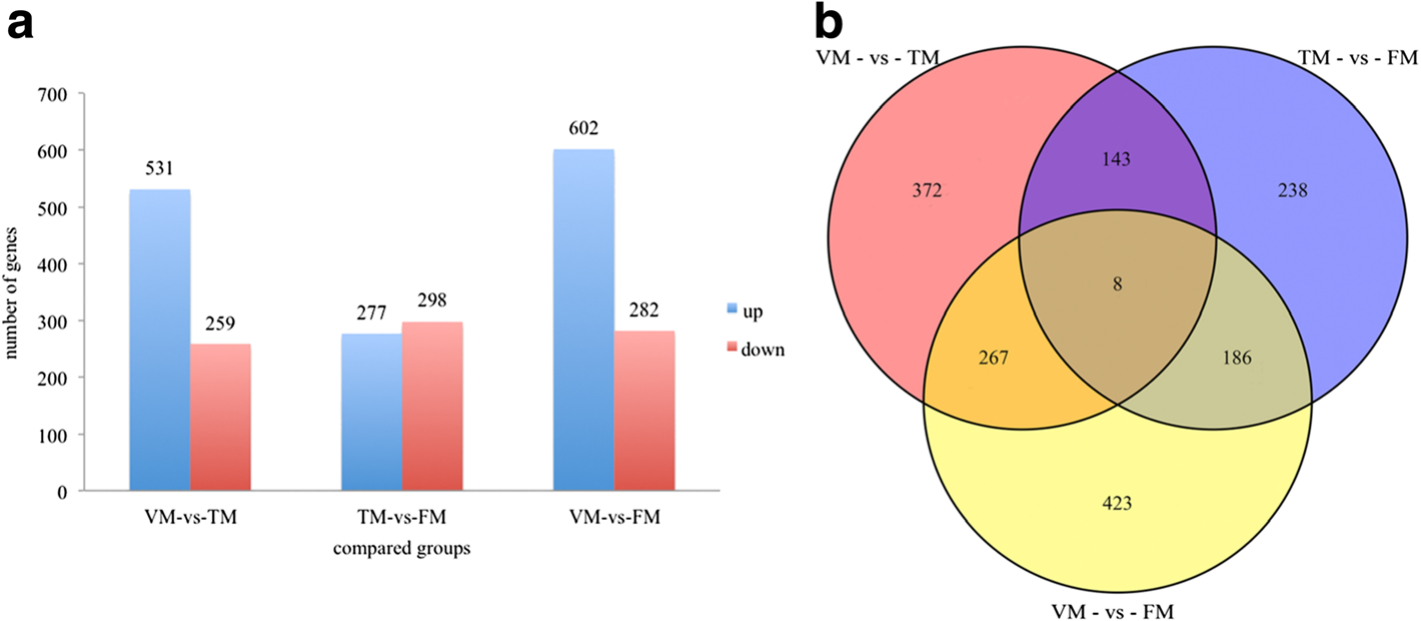
Numbers of differentially expressed genes in each comparison. **a** Numbers of DGEs in each comparison; numbers on column show the quantity of upregulated (*blue*) and downregulated (*red*) genes. **b** Veen diagram analyses of differentially and stage-specific expression genes per comparison

To confirm DGE at the three floral transition stages (VM, TM, and FM), seven cDNA libraries were constructed (VM, TM, and FM stages with three, two, and two biological replicates, respectively). Based on these analyses, we identified 531 upregulated and 259 downregulated DEGs between VM and TM. Similarly, 277 upregulated and 298 downregulated DEGs and 602 upregulated and 282 downregulated DEGs, respectively, were obtained from TM to FM and VM to FM. (Fig. 5a and Additional file 7). The most DEGs were identified from VM to FM. A number of DEGs were not only specifically expressed between VM and TM, but between TM and FM or between VM and FM, while a large number of DEGs were phase-specific. There were 372, 238, and 423 DEGs for VM versus TM, TM versus FM, and VM versus FM, respectively (Fig. 5b).

### Functional enrichment of DEGs

**Fig. 6:**
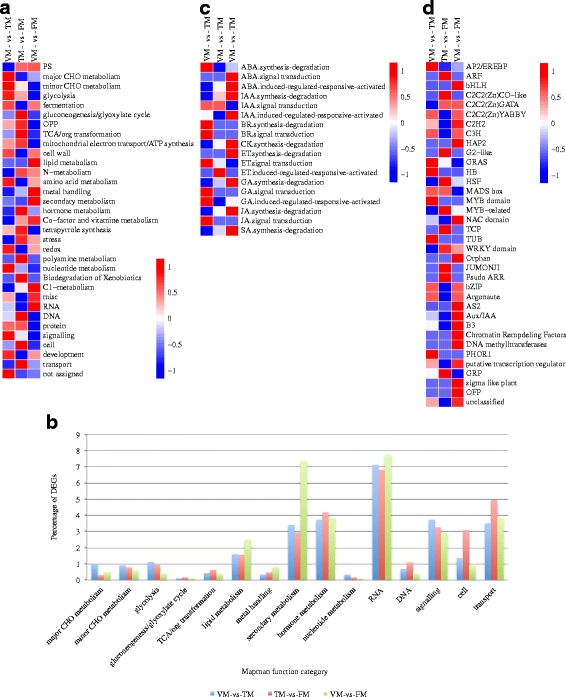
Enrichment of functional categories for DEGs in three compared groups. **a** Enrichment of functional categories for DEG lists. **b** The percentage of each functional category for DEGs. **c** Enrichment of hormone metabolism subcategories. **d** Enrichment of transcription factor subcategories

All DEGs of the three groups were assigned to MapMan functional categories. The DEGs between VM and TM were mainly enriched for RNA, hormone metabolism, signaling, cell, and secondary metabolism functions (Fig. 6a and b). DEGs that distinguished TM and FM as well as VM and FM were, respectively, mainly enriched for RNA, transport, and hormone metabolism and RNA, secondary metabolism, hormone metabolism, and transport. Regarding hormone metabolism categories, the DEGs from VM to TM were mainly associated with abscisic acid synthesis degradation, auxin signal transduction, ethylene signal transduction, gibberellin signal transduction, and gibberellin induced-regulated-responsive-activated (Fig. 6c and Additional file 8). In addition, in the comparison between VM and TM, the main differentially expressed transcription factors were AP2/EREBP, GRAS, MYB domain, TUB, bZIP, and PHOR1 (Fig. 6d).

### DEGs specifically expressed at the floral induction stage

**Fig. 7:**
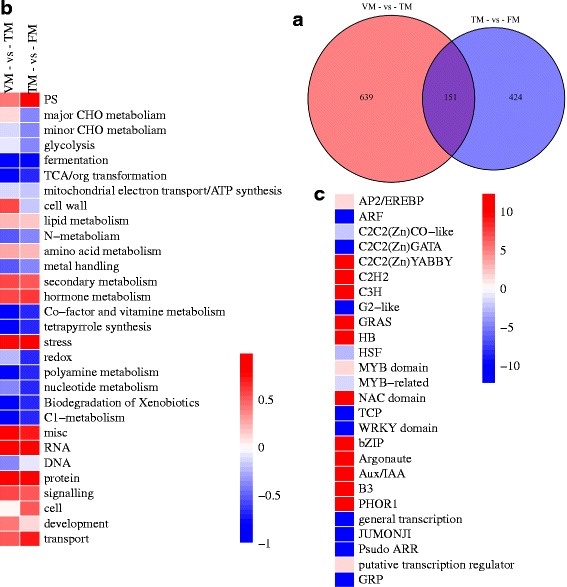
Analysis of stage-specific expression genes. **a** Venn diagram analysis of the number of stage-specific expression genes between VM - vs - TM and TM - vs - FM. **b** Enrichment of functional categories for DEG belonging to VM - vs - TM and TM - vs - FM, respectively. **c** Transcription factor families enriched in VM - vs – TM. The bar shows the log_10_ value of the number of enriched genes in B, while the bar shows the log_2_(VM-vs-TM)/(TM-vs-FM) value of the number of enriched genes in C

The DEGs between VM and TM may play a key role in floral induction. A total of 639 DEGs were specifically detected between VM and TM, while 424 DEGs were specifically detected between TM and FM, and 151 DEGs were shared between the two comparisons (Fig. 7a). The phase-specific DEGs were assigned to MapMan functional categories. The CHO metabolism, cell wall, hormone metabolism, signaling, and development categories were enriched in the analysis of specific DEGs between VM and TM (Fig. 7b). The major CHO metabolism category included genes encoding sucrose synthase (*SUS2*, c23831_g1; *SUS6*, c19920_g1), a pfkB-like carbohydrate kinase family protein (c19975_g1), and AGPase (c29462_g1). These genes were predominantly involved in the sucrose and starch metabolism pathways. In the development category and biosynthesis subcategory, genes encoding ALF (aberrant leaf and flower protein, c28547_g1), which is a transcriptional regulator, and the protein, which is a floral meristem identity gene involved in the transition from vegetative SAM to inflorescence meristems, were detected [13]. E3 ubiquitin-protein ligase *COP1*-like (c30945_g4) is involved in the photoperiod pathway. *SCL13* (scarecrow-like 13, c20369_g1) is a member of the *GRAS* gene family and is involved in the gibberellin signal transduction pathway. In the RNA regulation of transcription subcategory, the zinc finger protein *CONSTANS-LIKE* 16-like (c30323_g1) functions as the central regulator of the photoperiod pathway. The B3 domain-containing transcription factor *VRN1* (c32035_g1) is an *APETALA1/FRUITFULL* homolog, and activates flowering in a rhythmic manner. These genes were specifically differentially expressed between VM and TM and regulated floral induction. In the RNA category, the DEGs were associated with dozens of transcription factor families. The TFs enriched in the VM versus TM comparison were AP2/EREBP, C2C2 (Zn) YABBY, C2H2, GRAS, NAC domain, bZIP, AUX/IAA, and PHOR1 (Fig. 7c).

### GO analysis of differentially expressed genes

**Fig. 8:**
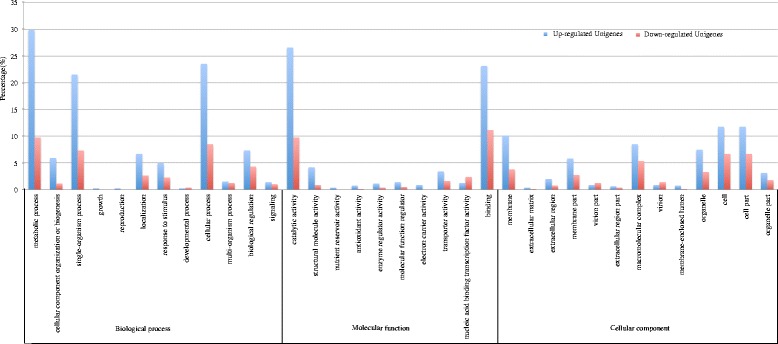
The GO classification of DEGs specifically expressed between VM and TM. The blue bar represents the percentage of up-regulated genes, while the red bar represents the percentage of down-regulated genes

Up- and downregulated DEGs between VM and TM were subjected to an enrichment analysis for GO annotation terms. In total, 791 DEGs were divided into three categories, cellular component, biological process, and molecular function. In the biological process category, the upregulated and downregulated DEGs were enriched for genes involved in metabolic processes, cellular processes, and single-organism processes. In the molecular function category, the upregulated and downregulated DEGs were mainly enriched for catalytic activity and binding. In the cellular component category, the upregulated DEGs were mainly associated with the cell, cell part, membrane, and macromolecular complex subcategories, while the downregulated DEGs were enriched for the cell, cell part, and macromolecular complex subcategories (Fig. 8).

### KEGG pathway enrichment analysis of DEGs

To characterize the expression profile of the 1,637 DEGs, the expression data υ (from VM to TM and TM to FM) were normalized to 0, log_2_^(TM/VM)^, and log_2_^(FM/VM)^. In total, 1,160 DEGs clustered into eight profiles based on an analysis using Short Times-series Expression Miner (STEM) (Additional file 9) [14]. The DEGs between VM and TM were mainly associated with floral transition of the rose and genes that belonged to profiles 3 and 4 showed no significant difference from VM to TM stage. Therefore, profiles 0, 1, 2, 5, 6, and 7 were chosen for subsequent analyses. Profiles 6 and 7 were upregulated and contained 307 and 134 DEGs, respectively; profiles 0 and 1 were downregulated and contained 49 and 103 DEGs, while profiles 2 and 5 contained 107 and 183 DEGs, respectively (Additional file 9).

All DEGs that belonged to profiles 0, 1, 2, 5, 6, and 7 were subjected to a KEGG pathway enrichment analysis. The DEGs were assigned to 85 KEGG pathways. The top 10 pathways are shown in Additional file 10. Partial KEGG pathways associated with plant floral transition are listed in Table 2. Three out of 133 unigenes in profile 5 (2.26%), 2 out of 49 unigenes (4.08%) in profile 7, and 1 out of 19 unigenes (5.26%) in profile 2 belonged to the circadian rhythm–plant pathway, while in profiles 0, 1, and 6, no unigene belonged to this pathway. Five our of 49 unigenes (10.2%) in profile 7, and 1 unigene accounting for 2.7, 5.56, and 0.74% of genes, respectively, in profiles 0, 1, and 6 belonged to the plant hormone signal transduction pathway, while no unigene in this pathway was detected in profiles 2 and 5. In addition, 38 out of 133 unigenes (28.57%) in profile 5 belonged to the carbon metabolism pathway, and 15 out of 136 unigenes (11.03%) in profile 6 belonged to the starch and sucrose metabolism pathway.

**Table 2:** Partial KEGG pathways associated with rose floral transition

Pathway	No. of DEGs with pathway annotation							Pathway ID
	ALL profiles	Profile 0	Profile 1	Profile 2	Profile 5	Profile 6	Profile 7	
Carbon fixation in photosynthetic organisms	93(14.81%)	0(0.00%)	0(0.00%)	0(0.00%)	30(22.56%)	18(13.24%)	7(14.29%)	Ko00710
Photosynthesis	23(3.66%)	0(0.00%)	0(0.00%)	0(0.00%)	0(0.00%)	0(0.00%)	3(6.12%)	Ko00195
Pentose phosphate pathway	44(7.01%)	0(0.00%)	0(0.00%)	0(0.00%)	15(11.28%)	15(11.03%)	7(14.29%)	Ko00030
Spliceosome	52(8.28%)	12(32.43%)	3(16.67%)	0(0.00%)	6(4.51%)	1(0.74%)	0(0.00%)	Ko03040
Fructose and mannose metabolism	43(6.85%)	0(0.00%)	0(0.00%)	0(0.00%)	7(5.26%)	18(13.24%)	7(14.29%)	Ko00051
Photosynthesis – antenna proteins	9(1.43&)	0(0.00%)	0(0.00%)	0(0.00%)	1(0.75%)	0(0.00%)	1(2.04%)	Ko00196
Zeatin biosynthesis	3(0.48%)	1(2.7%)	0(0.00%)	0(0.00%)	1(0.75%)	1(0.74%)	0(0.00%)	Ko00908
Carbon metabolism	120(19.11%)	0(0.00%)	0(0.00%)	0(0.00%)	38(28.57%)	18(13.24%)	7(14.29%)	Ko01200
Circadian rhythm - plant	7(1.11%)	0(0.00%)	0(0.00%)	1(5.26%)	3(2.26%)	0(0.00%)	2(4.08%)	Ko04712
Plant hormone signal transduction	10(1.59%)	1(2.7%)	1(5.56%)	0(0.00%)	0(0.00%)	1(0.74%)	5(10.2%)	Ko04075
Starch and sucrose metabolism	23(3.66%)	0(0.00%)	0(0.00%)	0(0.00%)	4(3.01%)	15(11.03%)	2(4.08%)	Ko00500

### DGEs associated significantly with rose floral transition

**Fig. 9:**
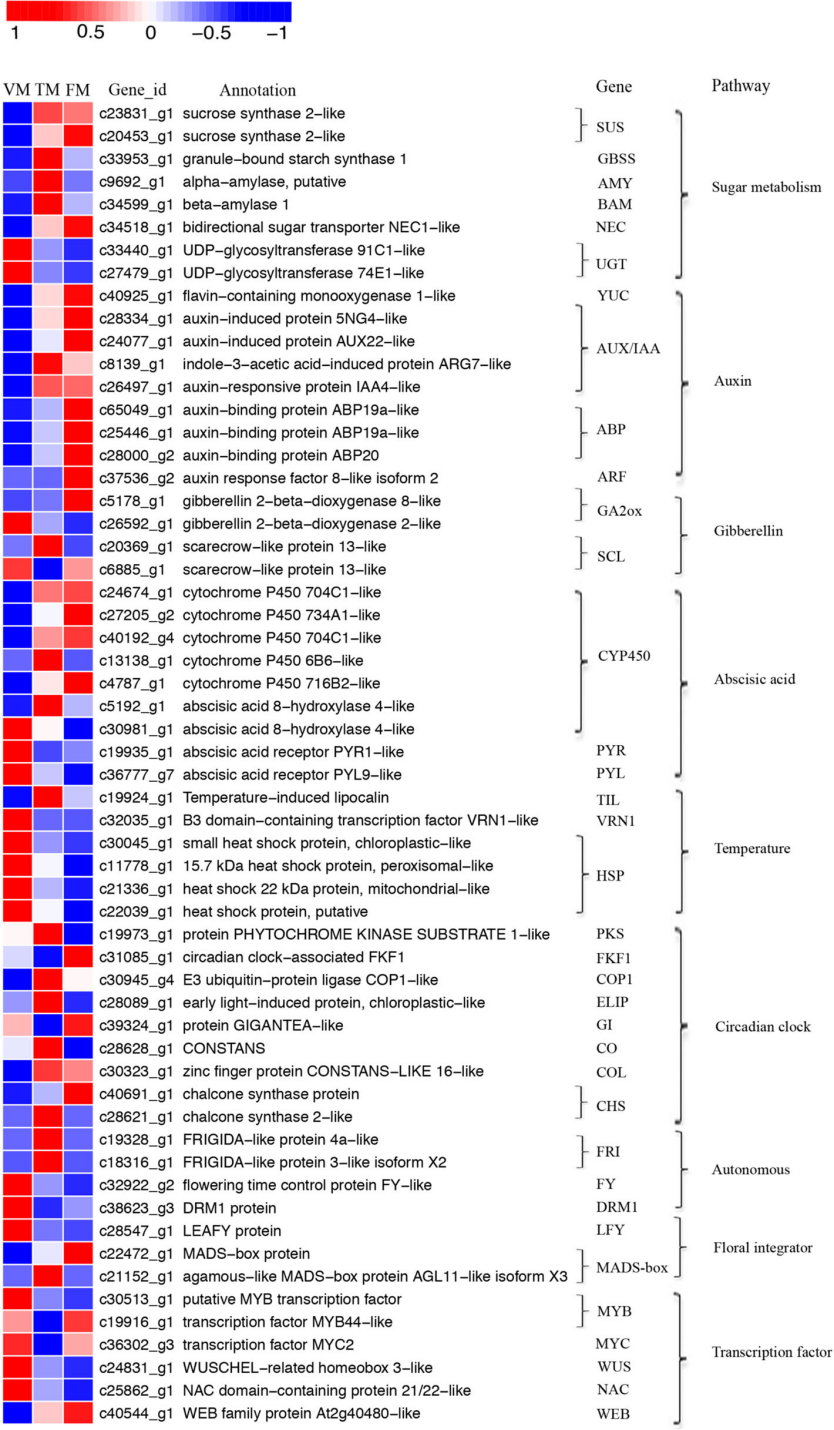
Heat map diagram of DEGs associated with floral transition in the rose. Data for gene expression levels were normalized to the Z-score

Table 3 shows the number of DEGs that were likely associated with rose floral transduction. A total of 32 unigenes were mainly involved in plant hormone signal transduction, photoperiod (circadian rhythm), sugar metabolism, temperature, autonomous pathway, and flowering activation and repression (Table 3 and Fig. 9).

**Table 3:** Number of DEGs associated with rose floral transduction

Components	All profiles	Profile 0	Profile 1	Profile 2	Profile 5	Profile 6	Profile 7
Flowering activators							
*FY*	1	1	0	0	0	0	0
*DRM1*	1	0	1	0	0	0	0
*LFY*	1	0	1	0	0	0	0
*MADS*	1	0	0	0	0	0	1
*AGL11*	1	0	0	0	1	0	0
*FRI*	2	0	0	0	2	0	0
*TIL*	1	0	0	0	0	1	0
Sugar metabolism							
*SUS2*	2	0	0	0	0	2	0
*NEC1*	1	0	0	0	0	1	0
*AMY*	1	0	0	0	1	0	0
Circadian clock pathway							
*CO*	1	0	0	0	0	0	0
*COL16*	1	0	0	0	0	1	0
*FKF1*	1	0	0	1	0	0	0
*ELIP*	1	0	0	0	1	0	0
*CHS*	2	0	0	0	1	0	1
Auxin							
*YUC*	1	0	0	0	0	0	1
*AUX/IAA*	3	0	0	0	0	2	1
*SAUR*	1	0	0	0	0	1	0
*ABP*	3	0	0	0	0	0	3
*ARF*	1	0	0	1	0	0	0
Gibberellin							
*GA2ox*	2	0	0	0	0	0	1
Abscisic acid							
*CYP707A*	2	1	0	0	0	1	0
*PYL*	1	1	0	0	0	0	0

In the autonomous pathway, *FY* and *DRM1* control the flowering time and activate flowering [15], and were clustered in profile 0. Two DEGs belonging to profile 5 were annotated as *FRIGIDA* (*FRI*), which is expressed at low levels in the VM and FM stages, but is highly expressed in the TM stage. In addition, DEGs belonging to profile 7 and profile 5, respectively, were annotated as *MADS* and *AGL11*, which are also involved in rose floral transduction (Table 3).

In the photoperiod pathway, two DEGs were annotated as *CONSTANS*, which is centrally involved in the relationship between day length and flowering [16]. They were assigned to profile 6, and predominantly differed in the comparison between VM and TM. Additionally, the circadian clock-controlled gene *FLAVIN-BINDING, KELCH REPEAT, F-BOX 1 (FKF1)* was clustered in profile 2. Two DGEs annotated as *chalcone synthase* (*CHS*) were assigned to profile 5 and profile 7, and were upregulated between VM and TM. In total, 5 out of the 6 DEGs that clustered into the photoperiod pathway showed upregulation from VM to TM and induced rose floral transition (Table 3 and Fig. 9).

In the auxin signal transduction pathway, three DEGs were annotated as auxin-binding proteins (ABPs), clustered to profile 7, and showed upregulated expression patterns. Three DEGs encoded auxin-induced protein (AUX/IAA), two of which belonged to profile 6 and one to profile 7; the expression level was relatively lower in the comparison between VM and TM (Fig. 9). In addition, one DEG was annotated as indole-3-acetic acid-induced protein (SAUR) and clustered in profile 6 (Table 3). Only one DEG was annotated as auxin response factor (ARF), which is dissociated by AUX/IAA. DEGs in the auxin signal transduction pathway positively regulated the rose floral transduction.

In the gibberellin biosynthesis pathway, only one DEG belonging to profile 7 was annotated as gibberellin 2-beta-dioxygenase (*GA2ox*), which catalyzes the 2-beta-hydroxylation of gibberellin precursors, rendering them unable to be converted to active GAs (Table 3). In contrast, *PYL9*, associated with the abscisic acid signal transduction pathway, belonged to profile 1 and showed a gradual decline during the floral transition process (Fig. 9). In addition, two DEGs belonging to profile 0 and profile 6 were annotated as *CYP707A*, which affects ABA levels.

In the sugar metabolism pathway, two DEGs belonging to profile 6 were annotated as sucrose synthase (*SUS*), and one DEG was annotated as bidirectional sugar transporter (*NEC*), belonging to profile 6. In addition, *AMY*, which is associated with starch metabolism, was classified as belonging to profile 5 (Table 3). These four DEGs showed similar patterns of upregulation between VM and TM, and positively regulated rose floral transduction (Fig. 9).

### Confirmation of unigene expression using real-time quantitative reverse transcription PCR

**Fig. 10:**
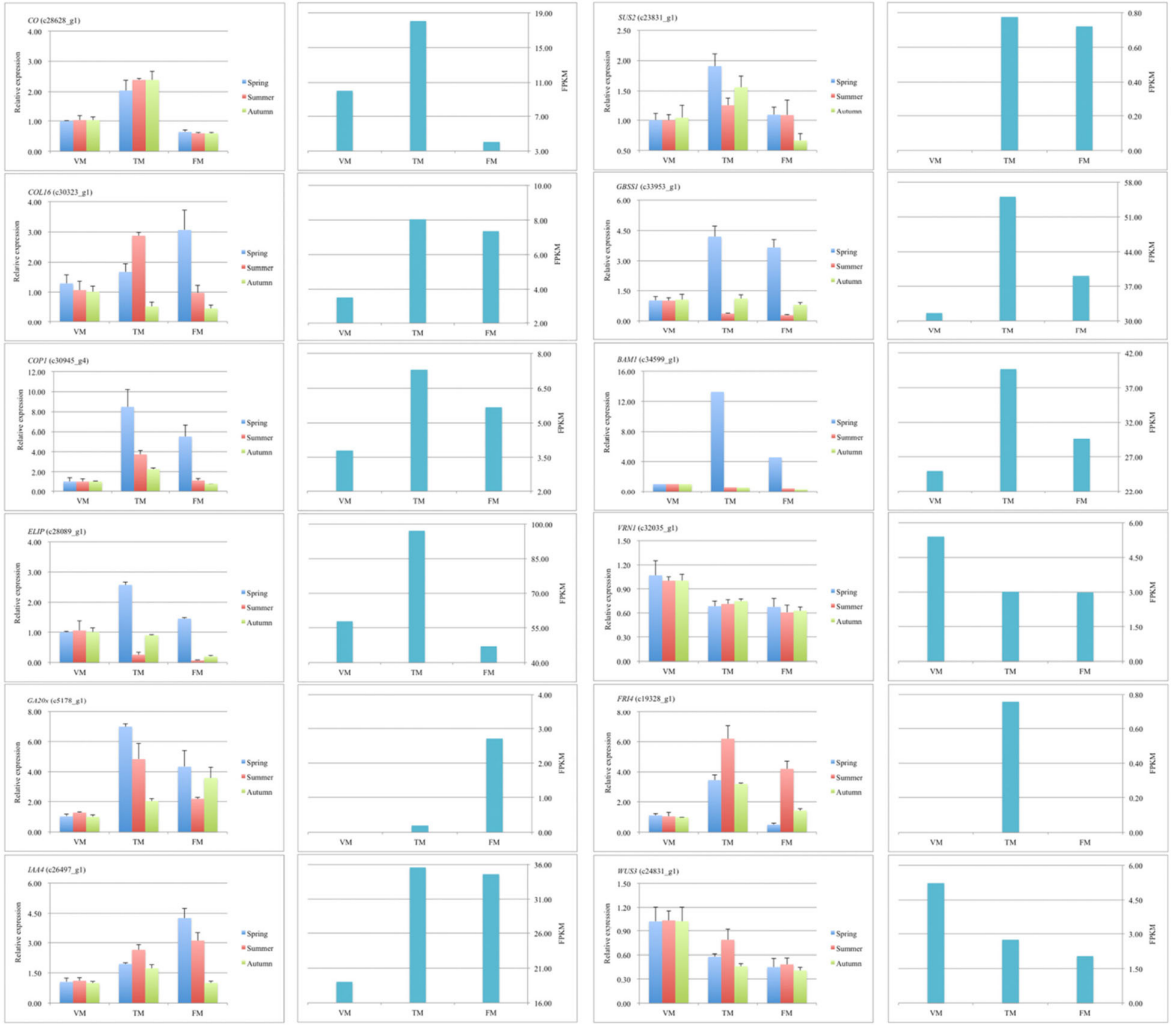
Candidate unigene expression levels revealed via RT-qPCR (the first and third column) and RNA-seq (the second and forth column). Data from RT-qPCR are means of three replicates and bars represent SE, data from RNA-seq are means of the replicates

**Fig. 11:**
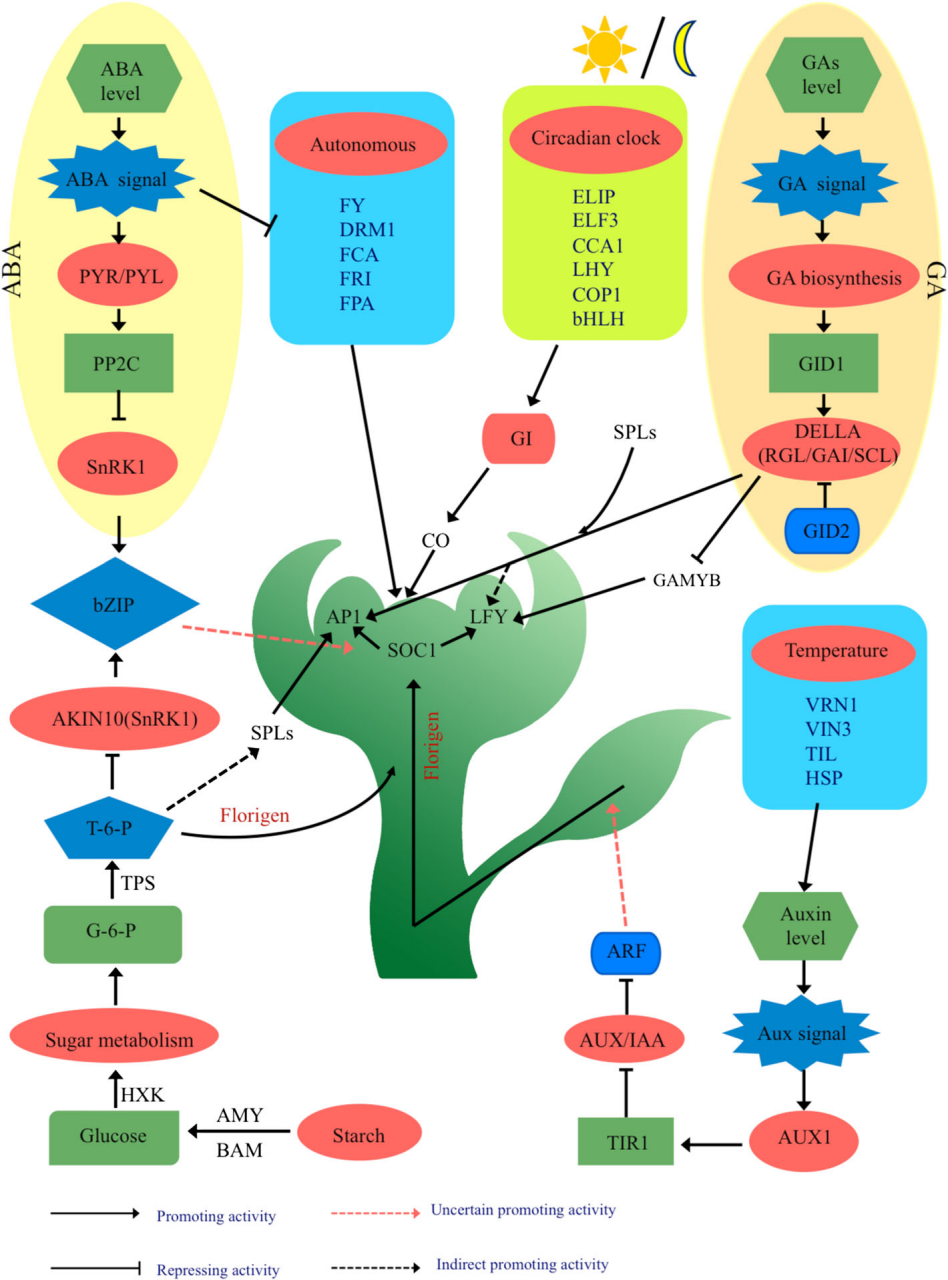
Hypothetical model for the regulatory networks of floral transition in the rose

To verify the accuracy and reproducibility of the transcriptome analysis, gene-specific primers were designed for 19 DEGs (Additional file 11). *R. chinensis* ‘Old Blush’ can flower year-round in favorable conditions (e.g., with respect to temperature and photoperiod); accordingly, we obtained RNA samples from the spring, summer, and autumn as templates, and validated the selected genes at the VM, TM, and FM stages. The expression profiles of the majority of candidate unigenes in the spring, summer, and autumn, based on RT-qPCR, were consistent with the RNA-seq results (Fig. 10 and Additional file 12). Additionally, the expression patterns for *COL16* (c30323_g1), *ELIP* (C28089_g1), *CHS* (c40691_g1), *BAM1* (c34599_g1), and *GBSS1* (c33953_g1) based on RT-qPCR were consistent with the sequencing results. However, the expression levels revealed by RT-qPCR in the summer and autumn samples were inconsistent with the results obtained for spring samples using RNA sequencing. In the autumn, *COL16*, *ELIP*, and *CHS* were downregulated during the floral transition. Similarly, *GBSS1* and *BAM1* were downregulated from VM to FM in the summer and autumn. Overall, these candidate DEGs synergistically regulate the floral transition in the rose. Based on these results, a hypothetical model for the regulatory networks involved in the rose floral transition in response to exogenous and endogenous cues was proposed, and is summarized in Fig. 11.

### Discussion

Flowering is a crucial developmental stage in the plant life cycle, especially for ornamental flowing plants. *R. chinensis* ‘Old Blush’ can flower continuously year-round, this trait is important for modern commercial roses and improves the yield and viewing time of modern roses. In this study, we investigated the complex molecular mechanisms underlying floral transition in *R. chinensis* ‘Old Blush’. Characterizing the transcript profiles at these three developmental stages provides a basis for identifying candidate genes regulating the floral transition in the rose, which could improve our overall understanding of regulatory networks involved in perennials.

### Functional enrichment of DEGs

All DEGs were annotated using MapMan software, and this analysis indicated that DEGs in the comparison between VM and TM were enriched for RNA, hormone metabolism, signaling, and transport functions (Fig. 6c), indicating that plant hormone (GAs, auxin, and ABA) signal transduction has a profound influence on the rose floral transition. In addition, genes in the major CHO metabolism category, including *SUS2* and *SUS6* affecting the sucrose and starch metabolisms, were enriched in the comparison between VM and TM (Fig. 6a), but not in the other two comparisons. We observed greater enrichment for genes in the secondary metabolism category for the TM versus FM comparison than the VM and FM comparison (Fig. 6a). DEGs in the flavonoid subcategory were enriched between TM and FM, suggesting that shoots in the FM stage have been in the flower development period. There were less DEGs in the comparison between TM and FM than in the other two groups, indicating that the TM stage and the FM stage may involve integral processes (Fig. 5b). Regarding the plant hormone signal transduction pathway, previous studies have verified that auxin, ABA, and GA signaling genes are involved in the floral transition process in rose [7, 9, 17]. In the present study, we furthermore found that genes involved in biosynthesis and degradation of these hormones expressed differentially during floral transition (Fig. 6c).

### Sugar signaling regulates flowering transition in the rose

Previous studies have demonstrated that sugars not only act as source of energy, but also as florigenic signals in plants [18, 19]. However, limited information is available about the regulatory role of sugar in flowering transition in the rose. The fluctuating sugar and starch levels in SAM may eventually be adjusted by alterations in the sugar flux or transitory starch to sugar [20]. In this study, gene expression patterns of sugar biosynthesis and transport were consistent with the content changes of sugars and starches during the floral transition process (Figs. 2 and 9 and Additional file 13), implying that sugar may act as a florigenic signal, triggering the transition from vegetative to reproductive state; similar results were observed for other plants [21]. Moreover, the increased levels of sugar during the floral transition also functioned as an osmotic pull for florigens, such as *SOC1*, *AP1*, and *FKF1*. Previous studies have indicated that there is a close relationship between flowering transition and sugar transporters [22]. Indeed, our results also showed that some flower integrators, i.e., *CO*, *COL16*, *SOC1*, *AP1*, and *AGL11*, are highly expressed in the flowering transition process, and similar patterns were observed for the expression of sugar transport-related genes and sugar contents. This suggests that sugar played a key role in inducing flowering by regulating the expression of flowering-related genes, such as *SOC1* and *AP1* (Additional file 13).

Starch biosynthesis and metabolism participate in the floral transition and promote flowering. Starch is the most important form of carbon reserve in plants. In particular, linear amylose is synthesized exclusively by *GSBB1* [23]. Interestingly, the photoperiodic factor *CO* regulates the expression of *GBSS1* and the composition of the starch granule [24]. Our results showed that the starch content decreased gradually from VM to FM stage (Fig. 2b). However, starch biosynthesis genes, such as *SS1-4*, *GSSS1*, *THI1*, and *SBPase*, were upregulated from VM to TM, and were slowly downregulated from TM to FM (Fig. 9 and Additional file 13), suggesting a higher transitory starch to sucrose ratio during the floral transition. Admittedly, the two genes *AMY* and *BAM1*, both participating in the starch degradation process, increased from VM to TM (Fig. 9), indicating that abundant soluble sugar was necessary for the flowering transition. Similar results were obtained in *Arabidopsis* [19]. However, the results of RT-qPCR for summer and autumn revealed that the expression patterns of *GBSS1* and *BAM1* had no obvious changes during floral transition. This suggests a decrease of the speed of biosynthesis and metabolism of starch.

T6P signaling pathway is also involved in flower induction by regulating vegetative growth and the transition to flowering. T6P is viewed as a proxy for carbohydrate status in plants [18]. Two genes, *TPS* and *TPP*, involved in T6P biosynthesis and degradation process, were upregulated and downregulated, respectively (Additional file 13), indicating that T6P was involved in the floral transition. Several studies have also reported that T6P and *AKIN10* (*SnRK1*) kinases play an opposite role in regulating the flowering transition in response to carbohydrate levels [25]. *AKIN10* decreased gradually from VM to FM (Additional file 13). Several SBP transcription factors, such as *SPL3*, *SPL4*, *SPL9*, and *SPL10*, which were floral activators, are inhibited by *miRNA156*. However, T6P represses the expression of *miRNA156* and indirectly activate *SPLs* to promotes the transition to flowering [26]. T6P also directly promotes FT, regulating the flowering transition via the T6P pathway [1]. Sugar signaling participates in the floral transition via multiple pathways. It will be interesting to study abnormal internal sugar levels in the rose to clarify these results.

### Hormone signaling mediates flowering transition in the rose

Plant hormones, as components of the flowering time syndrome, have been studied in model plants [27]. However, the intricate hormone regulatory networks underlying the floral transition in perennial plants remain unclear. In this study, we analyzed the gene expression profiles associated with hormone biosynthesis and signal regulatory pathways during floral transition process (Additional file 14).

GAs are important hormones involved in seed germination, floral induction, and development. Previous studies have demonstrated that GA plays a positive role in mediating the floral transition in *Arabidopsis* [5]. In contrast, GA negatively regulates the floral transition in woody plants, such as orange and apple [8, 28, 29]. However, a recent study showed that GA has dual, antagonistic roles in regulating the switch from vegetative to reproductive development. GA promotes the termination of vegetative development, but inhibits the floral transition in *Arabidopsis* [30]. In this study, the levels of GA_1_, GA_3_, and GA_4_ decreased sharply from VM to TM (Fig. 3c-e), and GA biosynthesis genes, such as *CPS*, *KO*, *GA20ox1*, and *GA3ox*, were downregulated from VM to TM (Fig. 9 and Additional file 14), while *GA2ox*, catalyzing bioactive GAs to inactive forms, was upregulated from VM to TM. These changes eventually contributed to low levels of GA, thus indicating that GA played a negative role in mediating the floral transition in the rose. The DELLA protein is a central node in GA signals, which interacted with *SPL3/4* to induce *AP1* and indirectly induce *LFY*, jointly inducing the floral transition [30]. Our results showed that DELLA proteins (GAI, RGL, and SCL4), *SPL4*, *AP1* were all upregulated from VM to TM (Fig. 9 and Additional file 14), indicating that DELLA proteins play vital roles in inducing the onset of flowering transition. *GAMYB*, a downstream component of GA response, binds to the promoter of *LFY* and enhances *LFY* expression; moreover, *GAMYB* can also transactivate a barley α-amylase promoter [31]. In the current study, α-amylase was upregulated from VM to TM (Fig. 9), suggesting that GA may interact directly with the starch metaolism or indirectly regulated the floral transition in the rose. It is worth mentioning, that naturally occurring GA_4_ is the most active GA during the floral induction process in *A. thaliana* [32]. However, the content of GA_3_ was much higher than GA_1_ and GA_4_ in the rose shoot apex, suggesting that the roles of GAs in rose floral transition are different from their roles in *A. thaliana*.

The role of auxin during the floral transition has been widely studied in the model plant, but less is known about its function in woody plants. Strawberry and rose belong to the same species, which can flower continuously. It has been reported that the spatial distribution of endogenous auxin and ABP protein gradually concentrated in the SAM during the floral transition process, indicating that auxin played a pivotal role in mediating the floral transition in the strawberry [33]. In the study, auxin contents and DEGs, *YUC*, and *ABP*, were synchronously upregulated from VM to TM (Figs. 3a and 9), while auxin-induced proteins, such as 5NG4 and SAUR, decreased (Additional file 14), suggesting that auxin-related genes involved in the floral transition in the rose. Similar observations have been reported in the seasonal rose *R. wichurana* [17]. The previous study demonstrated that auxin levels could be triggered by warm temperatures and elevated temperatures during the circadian clock window could promote flowering [34], indicating that an intricate regulatory relationship exists among the circadian clock, temperature, and auxin pathways. Further experiments are necessary to refine these relationships.

In the present study, ABA contents in the SAM of the rose were higher compared to other hormones, and decreased from VM to TM (Fig. 3b). The ABA synthesis gene *NCED* and its receptors, *PYL* and *PYR*, decreased during the floral transition process (Fig. 9 and Additional file 14), suggesting that genes involved in ABA signals may play an inhibitory role in the rose. However, Cui et al. [35] reported that ABA could promote *LcAP1* and trigger the floral transition in *Litchi chinensis. SnRK1* is involved in the sugar metabolism and also a key component of the ABA signalling pathway, which functions as a positive regulator in ABA signals [36], indicating that sugar may interact with ABA, consequently mediating floral transition. In addition, FCA is an ABA-binding protein, and the application of ABA affects the ratio between the long and short splice forms of FCA, and represses flowering [37].

### Flowering pathway in the rose during floral transition

The transition from vegetative to reproductive growth is a major physiological change in response to environmental (photoperiod, vernalization) and internal cues (autonomous, GA, sugar metabolism, and age) in *A. thaliana* [1]. *FKF1* is a circadian clock-controlled gene, which activates *CO* and interacts with *GI* [30]. In this study, *FKF1* was downregulated from VM to TM, while *CO* and *COL16* increased (Table 3, Additional file 15), indicating that photoperiod (circadian rhythm) positively mediated the rose floral transition.

Vernalization, i.e., the acceleration of flowering by extended exposure to cold conditions, epigenetically silences *FLC* via Polycomb proteins, which deposit the repressive histone mark H3K27me3 [38]. However, since *R. chinensis* ‘Old Blush’ can flower continuously throughout a year, we inferred that the flowering transition of rose is not influenced by vernalization. In fact, *FLC* does not express during floral transition in *R. chinensis* ‘Old Blush’ (not seen in data from RNA-seq and RT-qPCR). Interestingly, the key regulator, *FRI*, which has been reported to activate the expression of *FLC* in *A. thaliana* [39], was upregulated from VM to TM (Fig. 9). This difference requires further experiments to explore their potential functional diversification in regulating floral transition. Additionally, several genes expression in ambient temperature conditions were also differentially expressed, including *HSP70* and *HSF*, which were upregulated from VM to TM (Additional file 15). A previous study demonstrated that plants induced to flower by temperature and photoperiod cues exhibit high *HSP70* expression and gradual increases in temperature [40]. In the current study, *HSP70* increased from VM to TM (Additional file 15). The autonomous pathway is another important regulatory route including *FCA*, *FY*, *FPA*, *FLD*, *LD*, *DRM1* and so on, which function via a nonlinear hierarchy and generally promote flowering by repressing *FLC*, independently of vernalization [29]. During floral transition, *FY*, *FCA*, *FPA*, and *DRM1* were all downregulated from VM to TM, suggesting that autonomous pathway genes may promote flowering before the pre-floral stage.

The regulatory mechanism of flowering in the rose is complex, environmental and developmental factors converge towards a few floral integrator genes, such as *SOC1*, *CO*, *LFY*, and *AP1*, which irreversibly contributes to the transition from VM to FM. *TFL1* is downregulated from VM to FM and upregulated from TM to FM, which is consistent with its inhibitory role in the floral transition at the earliest stage, and its regulation of the inflorescence meristem during floral development [11]. The circadian rhythm involved connected feed-back loops, including *CCA1*, *LHY*, *GI*, *FKF1*, activated photoperiodic central gene, *CO*, which promoted flowering by activating *SOC1*. Subsequently, *SOC1* induced the expression of meristem identify regulators, i.e., *LFY* and *AP1*, to initiate flowering transition. In this case, *LFY* exhibited highest expression at the first stage, suggesting that *LFY* expression increased quickly before flowering commences, as observed in *Arabidopsis* [41]. A previous mapping study has already reported that the rose ortholog of the *A. thaliana* flowering time gene *SPY* is in close proximity to the *RB* locus [9]. In the study, *SPY* were upregulated during the flowering transition (Additional file 14), suggesting that *SPY* functions as a positive regulator for mediating flowering in the rose. Clearly, additional experiments are necessary to validate these proposed roles. In addition, *MiR156*-*SPL* regulates flowering in *A. thaliana* by interacting with age-dependent or sugar budget pathways. Furthermore, *SPL3*/*9* promotes the floral transition by activating MADS-box genes [26]. In the present study, several MADS-box family (*AGLs*, MADS-box protein, and *SVP*) and *SPL* family genes were jointly upregulated during the floral transition of the rose (Fig. 9), suggesting that genes of the MADS-box family play pivotal roles in the induction of the rose floral transition.

## Conclusions

Our results provide a comprehensive high-resolution characterization of gene expression profiles during the rose floral transition process. A number of DEGs were detected from the vegetative to reproductive growth stages, and these belonged to circadian rhythm, autonomous, hormone, and sugar metabolism pathways. A molecular mechanism was proposed in which many pathways collectively regulated the floral transition in *R. chinensis* ‘Old Blush.’ These results provide a valuable resource for studies in other closely related species with similar agricultural and ornamental value.

## Methods

### Plant material and experimental procedures

*R. chinensis* ‘Old Blush’ is a diploid continuously flowering rose; it contributed a key trait, recurrent flowering, to modern roses. Spring (January) samples were collected from a farm at Kunming Yang Chinese Rose Gardening Co., Ltd, Yunnan Province of China (24°45’N, 102°53’E) in 2015. Summer (July) and autumn (October) samples were obtained at the Xiao Tangshan nursery (40°09’N, 116°26’E), affiliated with Beijing Forestry University, (Beijing, China) in 2015. Leaves adjacent to the SAM were discarded as soon as possible, and mixed samples (~0.3 g) for each stage were collected from replicated plants, which were 1-year-old cutting seedlings. All samples were collected from 12:00 to 17:00, transferred immediately to liquid nitrogen, and stored at −80 °C until for for RNA-seq and RT-qPCR. Using paraffin sections, vegetative meristem (VM), pre-floral meristem (TM), and floral meristem (FM) spring samples were identified for transcriptome sequencing and sugar and hormone measurements, while summer and autumn samples were identified and then used for TR-qPCR. The spring samples of the VM, TM, and FM stages were used to construct nine libraries, named VM_YYF1, VM_YYF2, VM_YYF3, TM_YYF1, TM_YYF2, TM_YYF3, FM_YYF1, FM_YYF2, and FM_YYF3. Each stage had three replicates.

### Microscope observations

Shoots adjacent to leaves were cut and fixed quickly in FAA solution (formalin: acetic acid: 50% ethanol = 5/5/90 v/v). Fixed samples were dehydrated with a graded ethanol series (50 - 100%) embedded in paraffin, and sectioned into 8-μm slices (Lecia Microtome, Wetzlar, Germany). Dried sections were deparaffinized with xylene, hydrated in a decreasing ethanol series, and stained with Safranin and Fast Green. Slices were sealed using neutral gum and examined under a Scope A1 microscope (Zeiss, Jena, Germany). Figures were assembled using Adobe Photoshop (Adobe Systems, Mountain View, CA, USA).

### Measurements of sugar, starch, and hormone contents

The total sugar and starch contents were measured at three developmental stages, VM, TM, and FM. Approximately 0.3 g fresh weight of shoots was used for sugar and starch extraction, and sugar and starch contents were measured using the sulfuric acid-anthrone colorimetric method [42]. We used high-performance liquid chromatography-mass spectrometry (AB 5500, Beijing, China) to perform hormone identification and quantification, according to the protocol described in detail by Pan et al. [43].

### RNA extraction, quantification, and RT-qPCR analysis

Total RNA was extracted using EasySpin RNA Reagent (RN38; Aidlab Biotechnology, Beijing, China) according to the manufacturer’s protocol and treated with RNase-free DNase I (Takara, Dalian, China) to remove genomic DNA contamination. The specific primers for RT-qPCR were designed using PrimerQuest Tool (Additional file 12), and synthesized by Sangon Biotech Co., Ltd. (Beijing, China). Expression levels were normalized against the reference genes *RcActin* and *RcTCTP* [17]. RT-qPCR was conducted using the qTOWER 2.2 PCR System (Jena, Germany) and SYBR Green PCR Master Mix (TaKaRa, Japan). Each reaction was performed in a total reaction mixture volume of 20 μL containing 2 μL of first-strand cDNA as template. The amplification program was as follows: 3 min at 95 °C and 40 cycles of 10 s at 95 °C and 30 s at 60 °C. Each reaction was performed in three replicates. Expression levels of candidate genes were determined using the 2^−△△Ct^ method.

### RNA deep sequencing and library construction

The quality and quantity of RNA was determined using the NanoPhotometer^@^ spectrophotometer (IMPLEN, Westlake Village, CA, USA). Furthermore, the RNA concentration and integrity were assessed using the Qubit^@^ RNA Assay Kit and Qubit^@^ 2.0 Fluorometer (Life Technologies, Carlsbad, CA, USA) and the RNA Nano 6000 Assay Kit of the Agilent Bioanalyzer 2100 system (Agilent Technologies, Santa Clara, CA, USA), respectively. A total of 3 μg of RNA per sample was used to construct the cDNA library. The library was generated using the NEBNext® Ultra^TM^ RNA Library Prep Kit for Illumina® (NEB, Ipswich, MA, USA) following manufacturer’s instructions. Nine mixed RNA samples were subsequently used for cDNA library construction, and library quality was assessed using the Agilent Bioanalyzer 2100 system. The amplified fragments were sequenced using the Illumina HiSeq 4000 platform and 150-bp paired-end reads were obtained by Beijing Novogene Bioinformatics Technology Co., Ltd. (Beijing, China).

### De novo assembly and annotation

For the assembly library, raw data in fastq format were first processed using in-house Perl scripts. The raw reads were filtered by removing adapter sequences, reads containing poly-N sequences, and low-quality sequences. Clean reads were *de novo* assembled using Trinity [44], and the transcriptome reference database was obtained. All raw read data were deposited in the Genome Sequence Archive with the project ID PRJCA000258.

FPKM was used to obtain the relative expression levels [45]. A differential expression analysis of the two groups was performed using the DESeq R package (1.10.1). The resulting P-values were adjusted using Benjamini and Hochberg’s approach for controlling the false discovery rate. The DEGs were identified with a |fold change| ≥ 1.5 and a FDR < 0.05 between each comparison. The DEGs were annotated using the Mercator web tool [46] and then loaded to MapMan software for a functional enrichment analysis [47]. Additionally, gene expression data υ (from the VM to FM stage) were normalized to 0, log_2_^(TM/VM)^, and log_2_^(FM/VM)^, and DEGs were clustered by STEM [48]. Then Gene Ontology (GO) and Kyoto Encyclopedia of Genes and Genomics (KEGG) pathway analyses were performed [49, 50]. PCA analysis, venn diagrams, and hierarchical clustering heat maps in this study were generated using the gmodels, Venn diagram and Pheatmap packages in R based on the gene list and the levels of gene expression for each tissue type.

## Additional files

Additional file 1: Flowering transition process of *Rosa chinensis* ‘Old Blush’ revealed by paraffin sections. The process was divided into five stages: vegetative meristem, (VM); pre-floral meristem, (TM); sepal meristem, (SE); petal meristem, (PE); stamen meristem, (ST). (PDF 7305 kb)
Additional file 2: Length of unigene distributions for *R. chinensis* ‘Old Blush’. (PDF 100 kb)
Additional file 3: Characteristics of homology of rose unigenes. (a) Characteristics of homology search of rose unigenes. (b) E-value distribution of the top BLASTx hits against the Nr database. (PDF 176 kb)
Additional file 4: Number of DEGs annotated by GO. (PDF 156 kb)
Additional file 5: Percentage of DEGs enriched in KEGG annotation. (PDF 148 kb)
Additional file 6: Analysis of the repeatability of libraries using PCA. (PDF 1395 kb)
Additional file 7: Differentially expressed genes in each comparison. (XLSX 890 kb)
Additional file 8: Percentage of each hormone metabolism subcategories for DEGs. (PDF 141 kb)
Additional file 9: DEGs expression profiles (0–7) during the floral transition process. (PDF 72 kb)
Additional file 10: 10 top KEGG pathway. (PDF 54 kb)
Additional file 11: List of primers used in this study. (PDF 68 kb)
Additional file 12: Candidate unigene expression levels revealed via RT-qPCR (left side) and RNA-seq (right side). Data from RT-qPCR are means of three replicates and bars represent SE, data from RNA-seq are means of replicates. (PDF 6500 kb)
Additional file 13: Selection of sugar - related differentially expressed genes in rose. (PDF 117 kb)
Additional file 14: Selection of hormones - related differentially expressed genes in rose. (PDF 535 kb)
Additional file 15: Selection flower - related differentially expressed genes in rose. (PDF 725 kb)

## Abbreviations

ABA: Abscisic acid
AMY: Alpha-amylase
AP1: APETALA1
Aux: Auxin
AUX/IAA: Auxin-induced protein
BAM: Beta-amylase
CHS: Chalcone synthase
CO: CONSTANS
COL16: Constans-like 16
DEGs: Differentially expressed genes
ELIP: Early light-induced protein
FKF1: Flavin-binding, kelch repeat, f-box 1
FM: Floral meristem
GA: Gibberellin
GA2ox: Gibberellin 2-beta-dioxygenase
GBSS1: Granule-bound starch synthase 1
GO: Gene ontology
KEGG: Kyoto encyclopedia of genes and genomes
KING1/SnRK1: SNF1-related protein kinase regulatory subunit gamma-1-like
LFY: LEAFY
NCED: 9-cis-epoxycarotenoid dioxygenase
NEC: Bidirectional sugar transporter
PCA: Principal components analysis
PYL: Abscisic acid receptor PYL
SAM: Shoot apical meristem
SCL13: Scarecrow-like 13
SPS: Sucrose phosphate synthase
SS: Starch synthase
SUS: Sucrose synthase
TM: Pre-floral meristem
VM: Vegetative meristem
YUC1: Flavin-containing monooxygenase 1
