# Supplementary figures and images for: Transcriptome of the floral transition in Rosa chinensis ‘Old Blush’
Source: BMC Genomics. 2017 Feb 23;18:199. doi: 10.1186/s12864-017-3584-y (PMC5322666; doi:10.1186/s12864-017-3584-y)

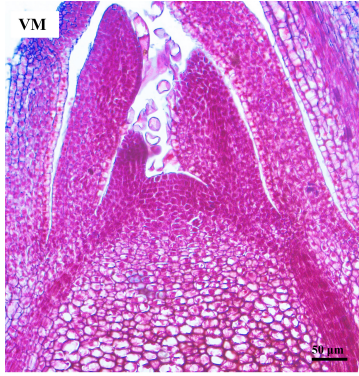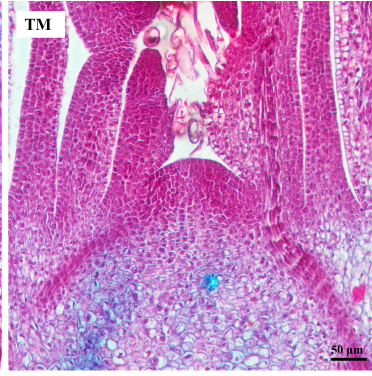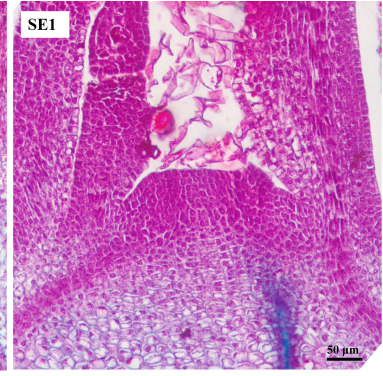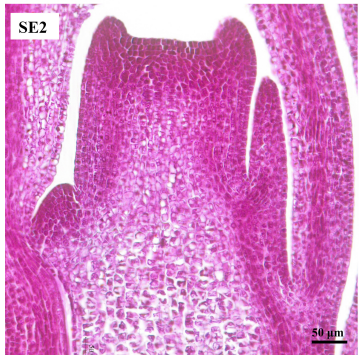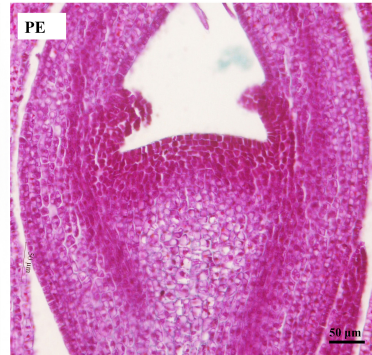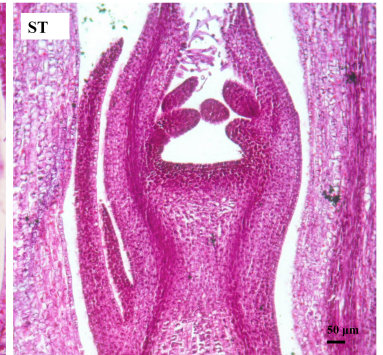

Supplement: Additional file 1: — Flowering transition process of Rosa chinensis ‘Old Blush’ revealed by paraffin sections. The process was divided into five stages: vegetative meristem, (VM); pre-floral meristem, (TM); sepal meristem, (SE); petal meristem, (PE); stamen meristem, (ST). (PDF 7305 kb) [file 12864_2017_3584_MOESM1_ESM.pdf]

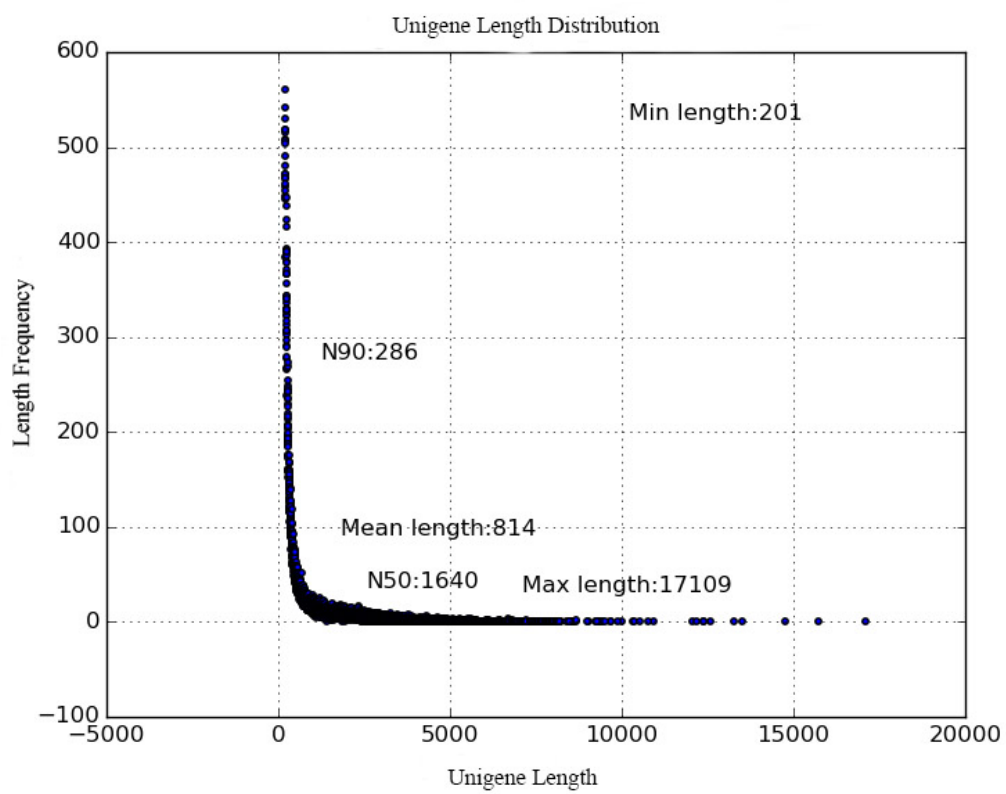

Supplement: Additional file 2: — Length of unigene distributions for R. chinensis ‘Old Blush’. (PDF 100 kb) [file 12864_2017_3584_MOESM2_ESM.pdf]

Gene Function Classification (GO)

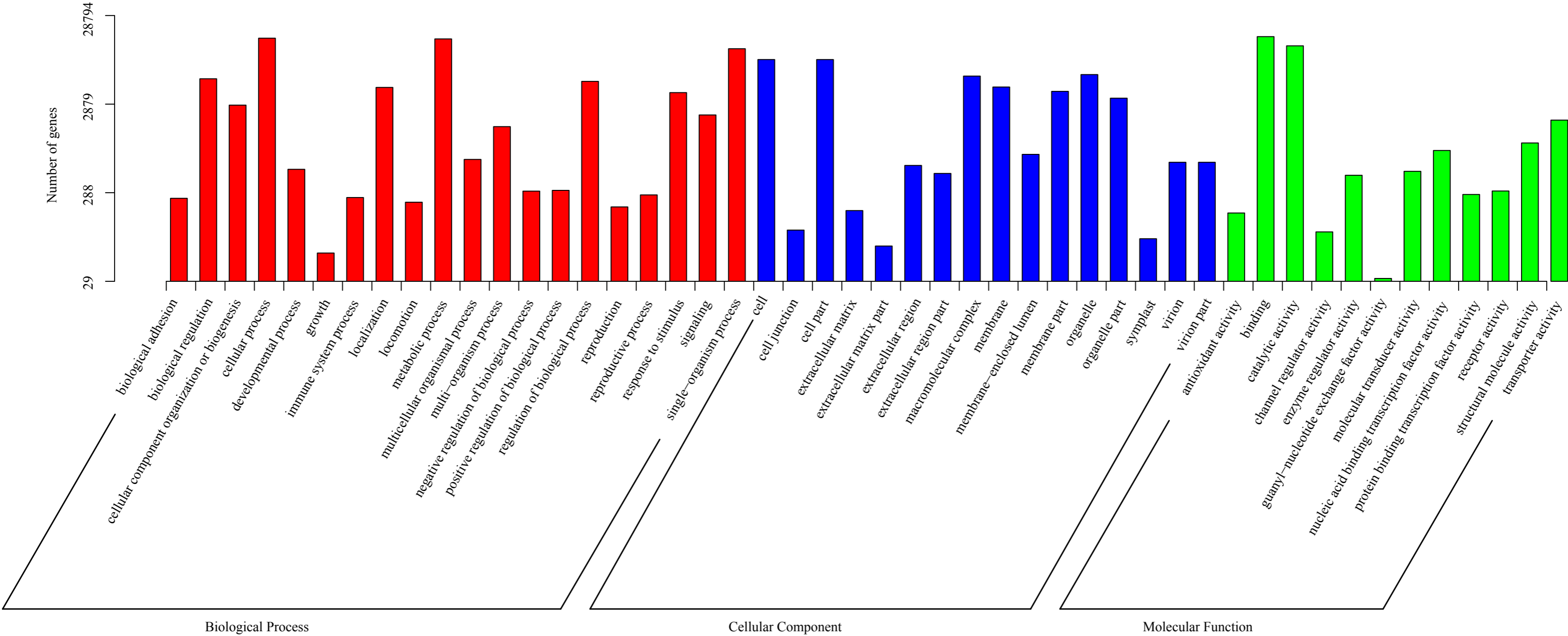

Supplement: Additional file 4: — Number of DEGs annotated by GO. (PDF 156 kb) [file 12864_2017_3584_MOESM4_ESM.pdf]

# KEGG Classification

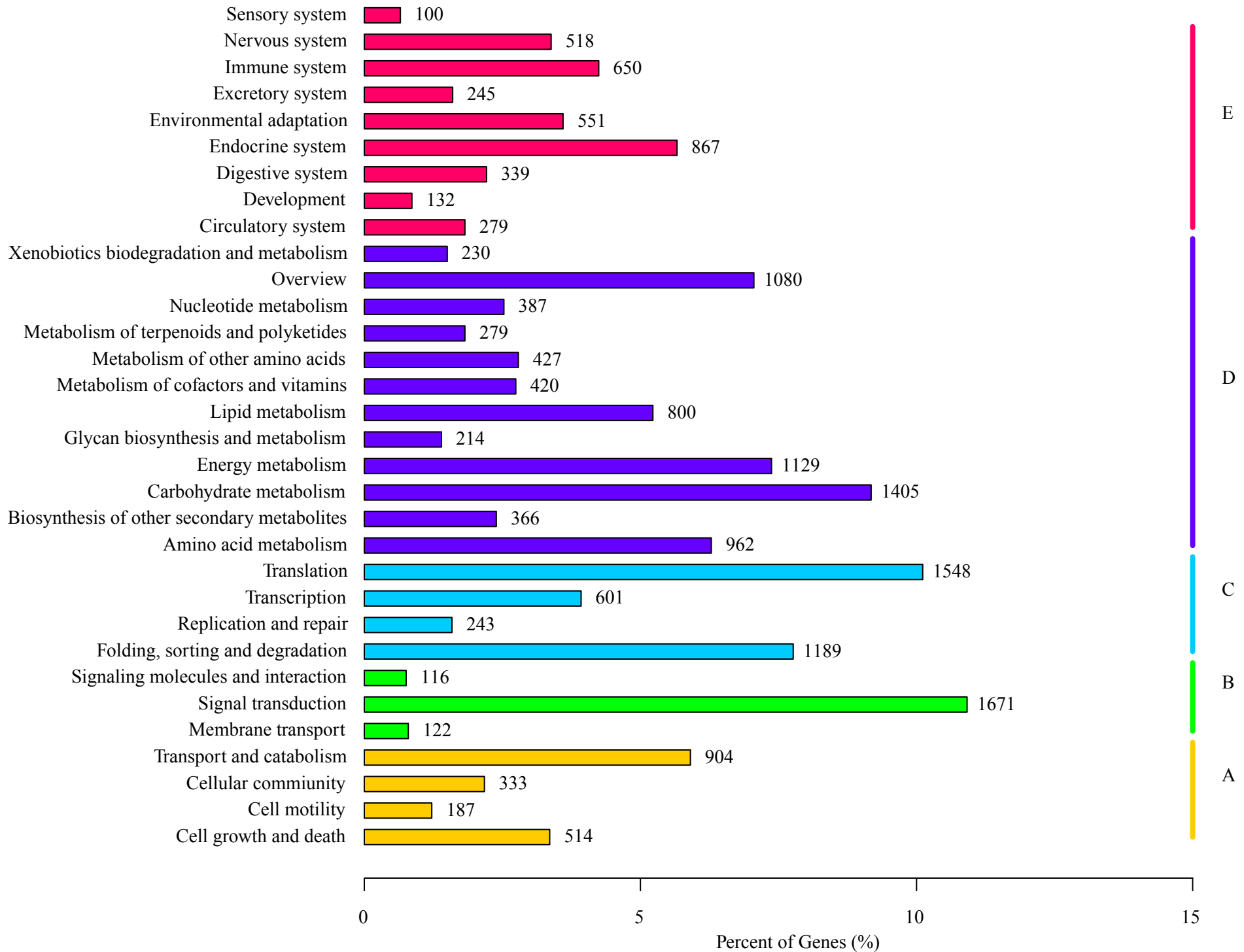

Supplement: Additional file 5: — Percentage of DEGs enriched in KEGG annotation. (PDF 148 kb) [file 12864_2017_3584_MOESM5_ESM.pdf]

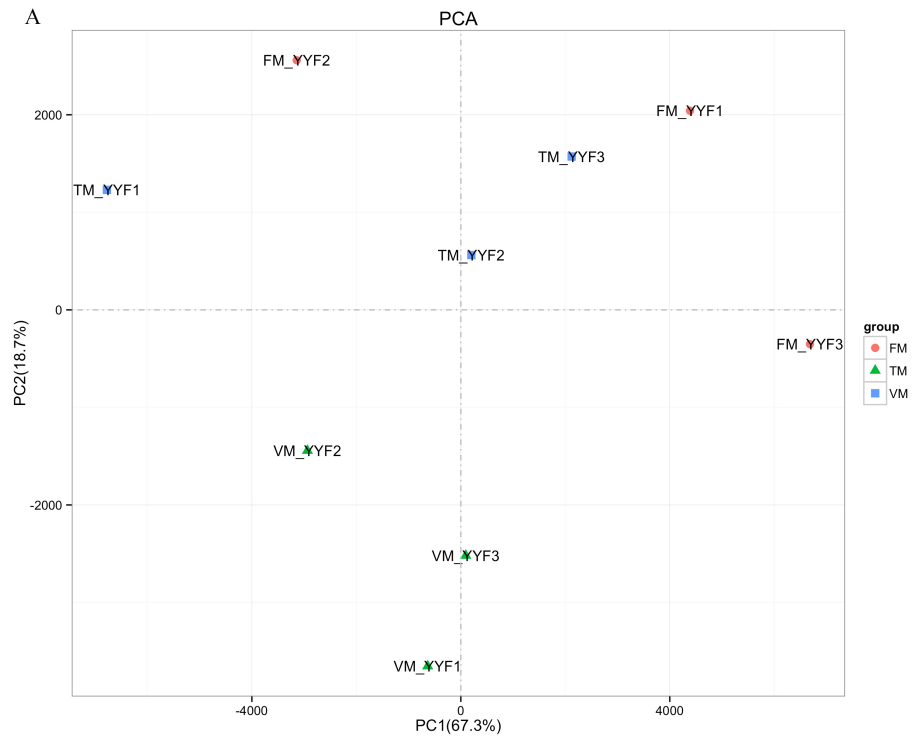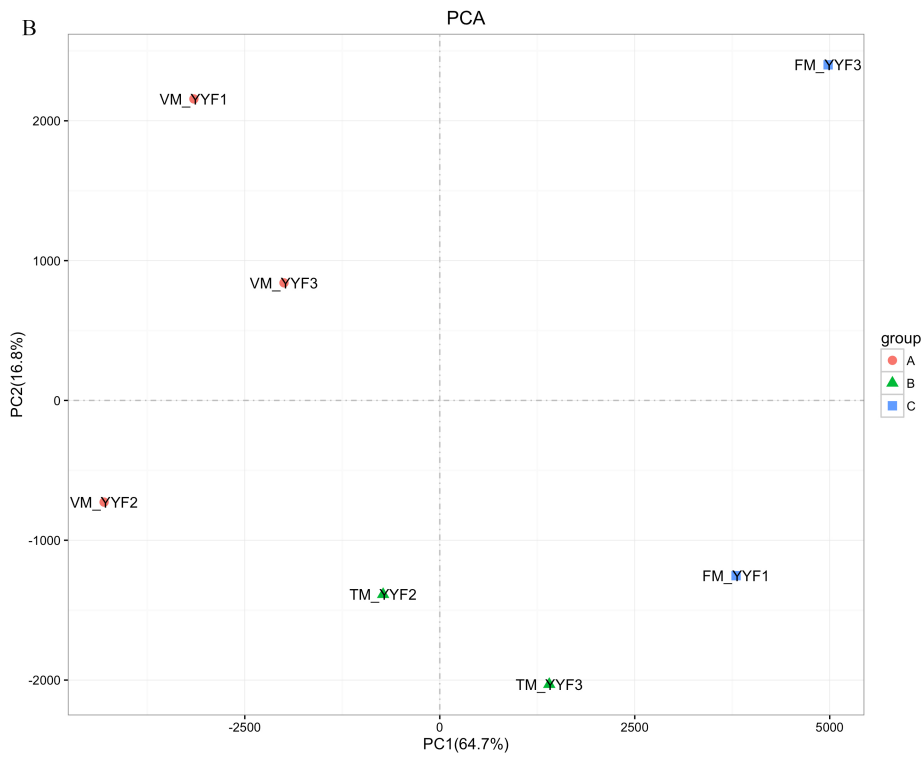

Supplement: Additional file 6: — Analysis of the repeatability of libraries using PCA. (PDF 1395 kb) [file 12864_2017_3584_MOESM6_ESM.pdf]

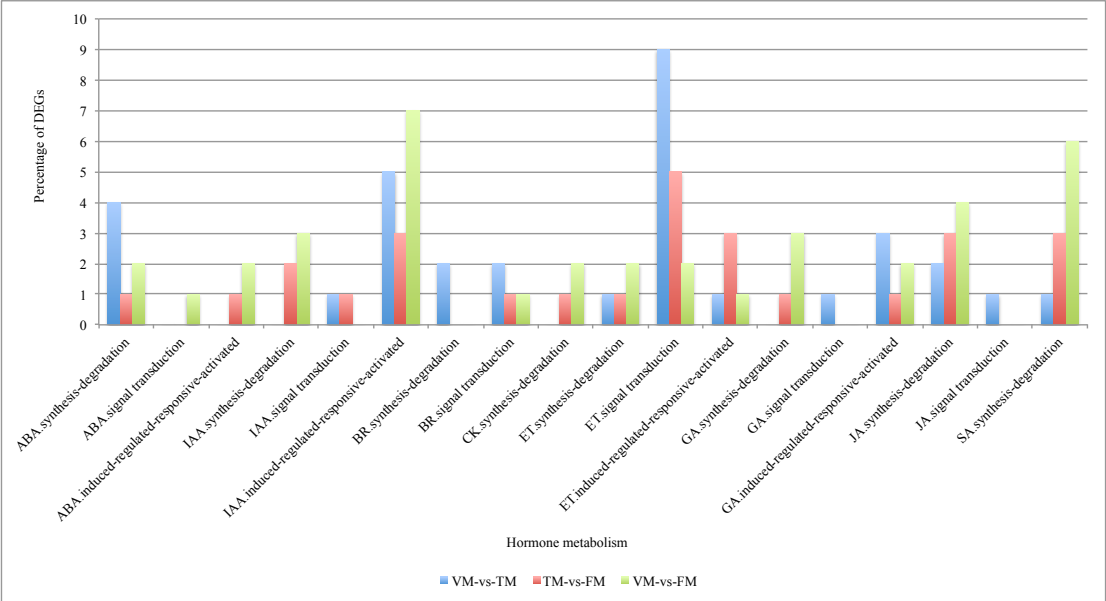

Supplement: Additional file 8: — Percentage of each hormone metabolism subcategories for DEGs. (PDF 141 kb) [file 12864_2017_3584_MOESM8_ESM.pdf]

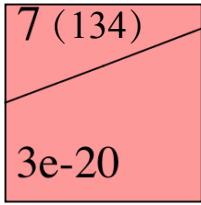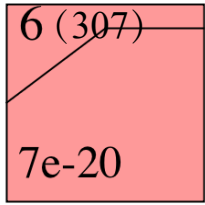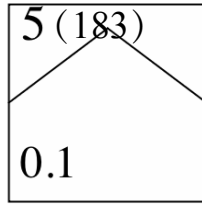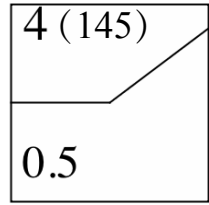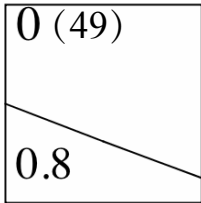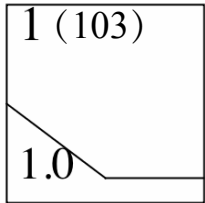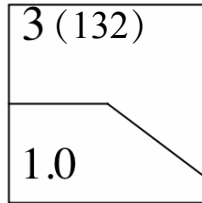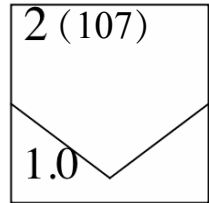

Supplement: Additional file 9: — DEGs expression profiles (0–7) during the floral transition process. (PDF 72 kb) [file 12864_2017_3584_MOESM9_ESM.pdf]

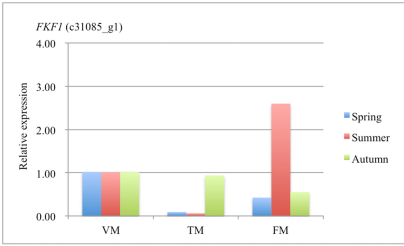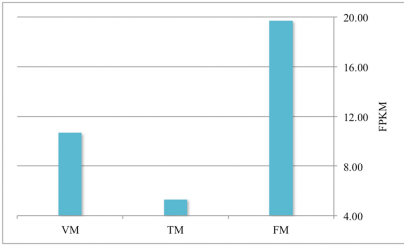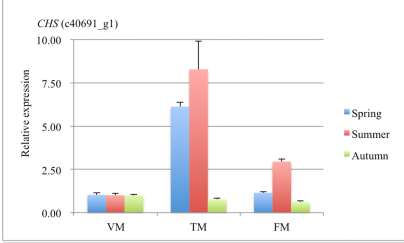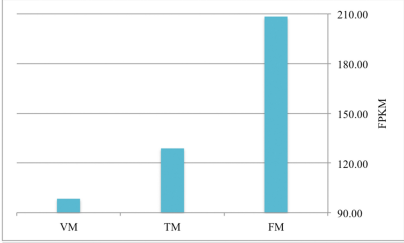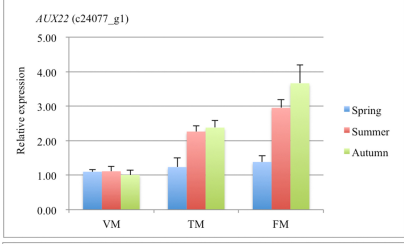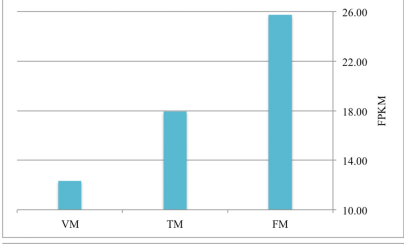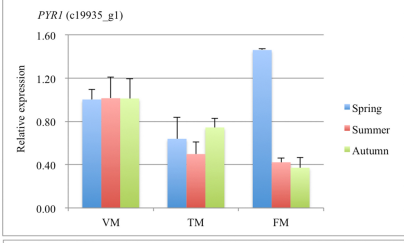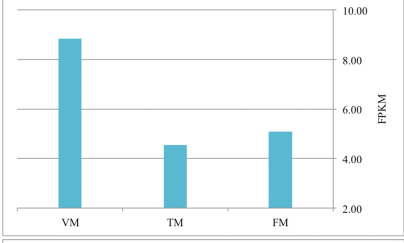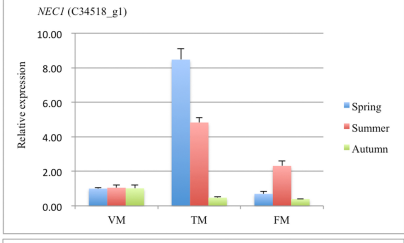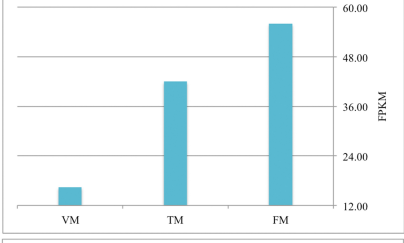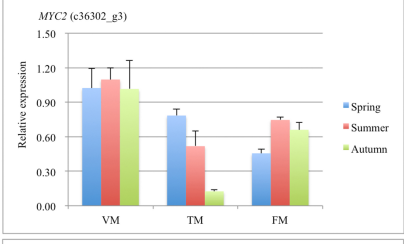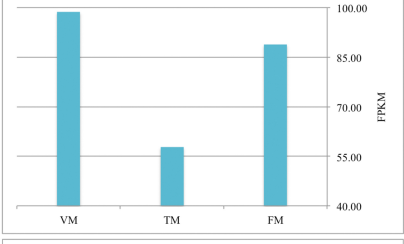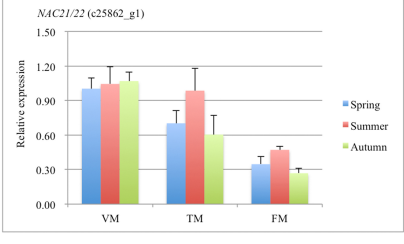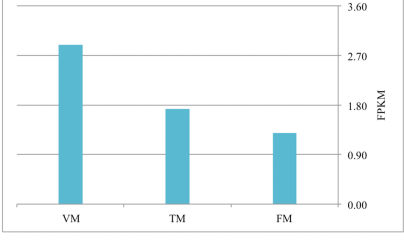

Supplement: Additional file 12: — Candidate unigene expression levels revealed via RT-qPCR (left side) and RNA-seq (right side). Data from RT-qPCR are means of three replicates and bars represent SE, data from RNA-seq are means of replicates. (PDF 6500 kb) [file 12864_2017_3584_MOESM12_ESM.pdf]
