# Supplementary material for: Transcriptome of the floral transition in Rosa chinensis ‘Old Blush’
Source: BMC Genomics. 2017 Feb 23;18:199. doi: 10.1186/s12864-017-3584-y (PMC5322666; doi:10.1186/s12864-017-3584-y)

A

Annotation	Number of Unigenes	Percentage (%)
Annotated in NR	38884	45.39
Annotated in NT	38438	44.87
Annotated in KO	15309	17.87
Annotated in SwissProt	30992	36.17
Annotated in PFAM	28330	33.07
Annotated in GO	28794	33.61
Annotated in KOG	15428	18.01
Annotated in all Databases	7263	8.47
Annotated in at least one Database	49671	57.98
Total Unigenes	85663	100

B

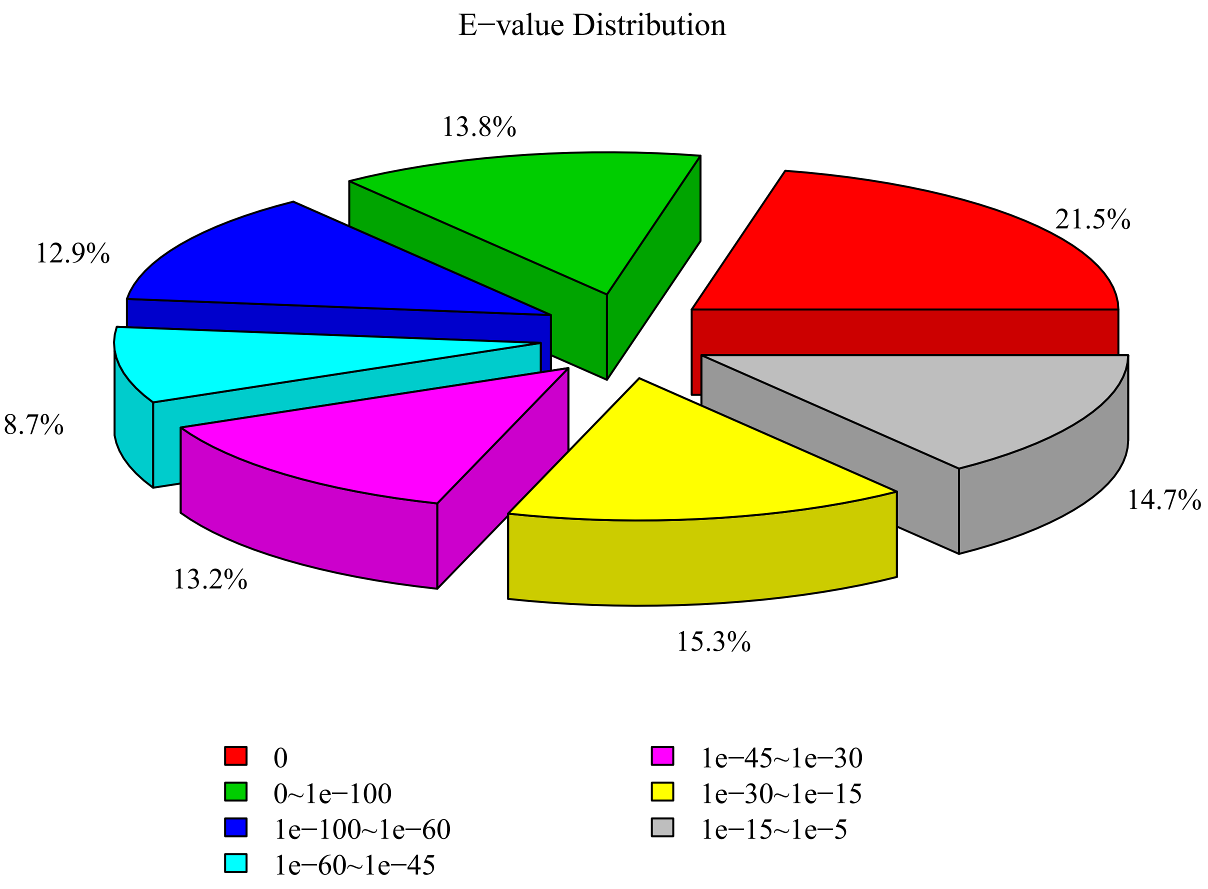

Supplement: Additional file 3: — Characteristics of homology of rose unigenes. (a) Characteristics of homology search of rose unigenes. (b) E-value distribution of the top BLASTx hits against the Nr database. (PDF 176 kb) [file 12864_2017_3584_MOESM3_ESM.pdf]
