# Supplementary material for: Transcriptome of the floral transition in Rosa chinensis ‘Old Blush’
Source: BMC Genomics. 2017 Feb 23;18:199. doi: 10.1186/s12864-017-3584-y (PMC5322666; doi:10.1186/s12864-017-3584-y)
Supplement: Additional file 10: — 10 top KEGG pathway. (PDF 54 kb) [file 12864_2017_3584_MOESM10_ESM.pdf]

Additional file 10. 10 top KEGG pathway

Pathway	DEGs with pathway annotation	Pathway ID
Carbon fixation in photosynthetic organisms	93(14.81%)	ko00710
Glyoxylate and dicarboxulate metabolism	67(10.67%)	ko00630
Photosynthesis	23(3.66%)	ko00195
Protein processing in endoplasmic reticulum	98(15.61%)	ko04141
Betalain biosynthesis	5(0.8%)	ko00965
Nitrogen metabolism	26(4.14%)	ko00910
Ubiquinone and other terpenoidquinone biosynthesis	18(2.87%)	ko00130
Isoquinoline alkaloid biosynthesis	23(3.66%)	ko00950
Pentose phosphate pathway	44(7.01%)	ko00030
Endocytosis	64(10.19%)	ko04144
