# Supplementary material for: Transcriptome of the floral transition in Rosa chinensis ‘Old Blush’
Source: BMC Genomics. 2017 Feb 23;18:199. doi: 10.1186/s12864-017-3584-y (PMC5322666; doi:10.1186/s12864-017-3584-y)
Supplement: Additional file 11: — List of primers used in this study. (PDF 68 kb) [file 12864_2017_3584_MOESM11_ESM.pdf]

Additional file 11. List of primers used in this study

**Genes**	**Gene_id**	**Forward (5' to 3')**	**Reverse (5' to 3')**
CO	c28628_g1	ATCTCCTCCCAGGTCGTATT	TTCAGCAGTTGGAGGAGTTC
COL16	c30323_g1	GGGATTACTGGATGGGACTAAAG	CCTCCTCCTCTTCAATCTTCAC
ELIP	c28089_g1	GAAAGCCAAAGCCACCAAAC	GTGTCAGCGTGGAGTCAAA
FKF1	c31085_g1	TAGGGACTTGGGTGGAAGA	GACTTGCATGAGTTGTGCTTAG
COP1	c30945_g1	GTCGTCCTTAGGCTGTTCAT	GACCCATCGGAAACTGTTCTA
CHS	c28621_g1	CTGTTCTTCGCCTCGCTAAA	CTGGCCCACAAGAGAATCAA
SUS2	c23831_g1	CAGATGAATCGCGTGAGGAA	CTCAACAACAGTCAAGCCAAAG
GBSS1	c33953_g1	TGACTGTTTCACCGCGTTAT	ACCGAACCATCTCAATCTTCTG
NEC1	c34518_g1	GTCGGTTCGTTCCTCGTTTA	ATGCCACTGAGTAGGGTAGA
BAM1	c34599_g1	AGGTGGAGGCTGGAATAGTA	GATAGACTTGGCTGAGGAAAGG
AUX22	c24077_g1	CGATCAATTCGCTGCTTACAAT	TACTGTCGTACCTCGTCTCTAC
IAA4	c26497_g1	ACTACTTGTGCCCTGGAAAC	CGTACCATGCAGATGAGCTAAA
GA2ox	c5178_g1	CCCAGAGATGTTTGGTCTAGTG	GCAACCCATTTGGAGTCTTTC
PYR1	c19935_g1	CGCATAGATCAGACCTGGAAAT	GATACGGTGGTCAGGTTGAAT
MYC2	c36302_g3	CGGAGAGACAGAGAAGAGAGAA	CTTGAGCTCGGTGATGTAAGAG
VRN1	c32035_g1	ACACACAAACATCTCCTTCCTC	GTCTGCACTTATGAGCCACTAC
FRI4	c19328_g1	GTCTCCCTCACACACTCAATAC	TGCTTCTGGTGAGTAAGCATAG
NAC21/22	c25862_g1	CACACTCCATACTACACACATACC	CACAGACTAAGCACAACCTTCT
WUS3	c24831_g1	ACCTAGCCAACTGCCTAATG	CACAACACACAGACCAAGAAAC
RcActin		TCAAGGATTGGTGGACTTCAGT	ACCAGAGAACAAGAATGCAAGC
RcTCTP		TTGGTCTTTGCCTACTACAAAGAGG	AAGCCAGTTGCTACTTCTTAGCACT
