# Supplementary material for: Transcriptome of the floral transition in Rosa chinensis ‘Old Blush’
Source: BMC Genomics. 2017 Feb 23;18:199. doi: 10.1186/s12864-017-3584-y (PMC5322666; doi:10.1186/s12864-017-3584-y)
Supplement: Additional file 13: — Selection of sugar - related differentially expressed genes in rose. (PDF 117 kb) [file 12864_2017_3584_MOESM13_ESM.pdf]

Additional file 13 Selection of sugar - related differentially expressed genes in rose

Rose genes Identification	Annotation	VM FPKM	VM Z-score (FPKM)	TM FPKM	TM Z-score (FPKM)	FM FPKM	FM Z-score (FPKM)
**Carbohydrate biosynthetic and metabolic process**							
c23831_g1	sucrose synthase 2-like (SUS2)	0.22	−1.15	0.78	0.67	0.72	0.48
c20453_g1	sucrose synthase 2-like (SUS2)	0.52	−1.08	0.97	0.20	1.21	0.88
c47951_g1	galactinol synthase 2-like isoform 1 (GolS1)	0.65	0.86	0.33	−1.10	0.55	0.24
c15639_g1	galactinol synthase 2-like (GolS2)	5.26	1.12	1.56	−0.33	0.4	−0.79
c27810_g1	galactinol synthase 2-like (GolS2)	124.74	−0.21	119.49	−0.88	134.81	1.09
c71288_g1	galactinol synthase 2-like isoform 2 (GolS2)	0.69	−0.19	0.44	−0.89	1.14	1.08
c67870_g1	galactose oxidase-like (GOase)	0.14	−0.45	0.39	1.15	0.1	−0.70
c23898_g1	glycosyl hydrolase family 1 (GH1)	0.11	−0.54	0.52	1.15	0.09	−0.62
c37263_g4	mannose-6-phosphate isomerase 2-like (MEE2)	15.39	1.12	7.21	−0.81	9.3	−0.31
c37505_g1	UDP-galactose/UDP-glucose transporter 4-like (ATUTR1)	12.8	−1.15	14.45	0.52	14.57	0.64
c39689_g2	probable galacturonosyltransferase 6-like (GAUT6)	104.94	−1.01	116.63	0.02	127.72	0.99
c33685_g2	galacturonosyltransferase 8-like (GAUT8)	91.97	1.05	78.52	−0.94	84.13	−0.11
c26651_g1	probable galacturonosyltransferase 10-like (GAUT10)	19.34	1.10	16.95	−0.85	17.67	−0.26
c34123_g1	probable galacturonosyltransferase-like 10-like (GAUT10)	47.79	1.02	18.7	−0.98	32.23	−0.05
c37573_g1	probable galacturonosyltransferase 12-like (GAUT12)	4.34	−1.14	6.81	0.73	6.38	0.41
c27816_g1	beta-1,3-glucanase (BG)	7.01	−0.89	9.7	−0.19	14.56	1.08
c34896_g2	SNF1-related protein kinase catalytic subunit alpha KIN10 (KIN10)	17.58	1.03	12.46	−0.96	14.76	−0.07
c28268_g2	SNF1-related protein kinase catalytic subunit alpha KIN10-like (KIN10)	49.96	0.95	47.57	0.08	44.48	−1.04
c33852_g1	SNF1-related protein kinase regulatory subunit beta-2-like (AKINB2)	13.72	−1.15	21.17	0.57	21.22	0.58
c26339_g2	SNF1-related protein kinase regulatory subunit beta-2-like (AKINB2)	31.22	0.47	29.64	−1.15	31.42	0.68
c26339_g1	SNF1-related protein kinase regulatory subunit beta-2-like (AKINB2)	0.12	−0.86	0.17	−0.24	0.28	1.10
c28577_g1	SNF1-related protein kinase regulatory subunit gamma-1-like (KING1/SnRK1)	10.62	0.54	10.77	0.61	6.8	−1.15
c27722_g1	SNF1-related protein kinase regulatory subunit gamma-1-like (KING1/SnRK1)	4.08	−1.15	6.34	0.50	6.54	0.65
c74776_g1	SNF1-related protein kinase regulatory subunit gamma-1-like (KING1/SnRK1)	0.3	1.15	0.16	−0.64	0.17	−0.51
c37976_g1	SNF1-related protein kinase regulatory subunit gamma-1-like (KING1/SnRK1)	12.75	0.94	10.43	0.12	7.11	−1.05
c33931_g1	UDP-arabinose 4-epimerase 1-like (MUR4)	31.74	−0.20	27.25	−0.89	40.07	1.08
c49723_g1	UDP-D-glucuronate 4-epimerase 1 (GAE1)	0	−0.58	0	−0.58	0.82	1.15
c72778_g1	UDP-D-glucuronate 4-epimerase 1 (GAE1)	0	−0.58	0	−0.58	0.24	1.15
c30664_g1	UDP-galactose transporter 1-like (UDP-GALT1)	35.88	−0.34	34.44	−0.78	40.65	1.13
c34426_g2	UDP-galactose transporter 2 (UDP-GALT2)	32.52	0.05	30.64	−1.02	34.16	0.98
c28804_g2	UDP-galactose transporter 2 (UDP-GALT2)	22.84	−1.00	23.99	0.00	25.14	1.00
c26604_g1	UDP-galactose/UDP-glucose transporter 3-like (UTR3)	91.07	0.95	74.64	0.10	52.25	−1.05
c33483_g1	UDP-glucose 4-epimerase GEPI48-like (EC 5.1.3.2)	16.12	−0.75	17.05	−0.38	20.84	1.13
c32031_g1	UDP-glucose 4-epimerase-like (EC 5.1.3.2)	21.89	0.28	24.41	0.83	15.47	−1.11
c30743_g1	UDP-glucose 6-dehydrogenase-like (EC 1.1.1.22)	34.56	−0.54	34.33	−0.62	39.61	1.15
c27658_g1	UDP-glucose 6-dehydrogenase-like (EC 1.1.1.22)	90.65	−1.06	104.27	0.14	113.23	0.92
c10176_g1	UDP-glucose pyrophosphorylase (UGPase)	0	−0.58	3.04	1.15	0	−0.58
c43055_g1	UDP-glucose pyrophosphorylase (UGPase)	0	−0.89	0.34	1.08	0.12	−0.19
c35304_g3	UDP-glucuronate 4-epimerase 1-like (GAE1)	91.64	0.96	60.35	−1.03	77.68	0.07
c21720_g1	UDP-glucuronate 4-epimerase 3-like (GAE3)	19.82	−1.00	23.13	1.00	21.47	−0.00
c29446_g1	putative UDP-glucose glucosyltransferase-like (GUX)	0.63	−1.00	0.85	0.00	1.07	1.00
c38633_g3	UDP-glucuronic acid decarboxylase 1-like (USX1)	108.11	−0.80	115.58	−0.32	137.79	1.12
c23016_g1	UDP-glucuronic acid decarboxylase 1-like (USX1)	0.22	−1.14	0.45	0.73	0.41	0.41
c34407_g3	UDP-glucuronic acid decarboxylase 1-like (USX1)	61.2	0.95	53.15	−1.04	57.74	0.09
c35359_g2	UDP-sugar-dependent glycosyltransferase 52-like (UGT52)	10.34	−0.57	11.24	1.15	10.33	−0.59
c39461_g1	UDP-sugar-dependent glycosyltransferase 52-like (UGT52)	9.68	−0.06	8.87	−0.97	10.66	1.03
c35359_g1	UDP-sugar-dependent glycosyltransferase 52-like (UGT52)	0.74	1.10	0.11	−0.86	0.31	−0.24
c62117_g1	UDP-glycosyltransferase 73B3-like (UGT73B3)	2.95	−1.02	3.78	0.03	4.53	0.98
c73702_g1	UDP-glycosyltransferase 73B3-like (UGT73B3)	1.62	−0.68	4.16	1.15	1.92	−0.47
c40863_g3	UDP-glycosyltransferase 73C5-like (UGT73C5)	96.77	0.06	111.46	0.97	79.34	−1.03
c34021_g1	UDP-glycosyltransferase 74B1-like (UGT74B1)	5.45	−0.90	8.01	−0.17	12.39	1.08
c15731_g1	UDP-glycosyltransferase 74B1-like (UGT74B1)	1.74	−0.81	3.09	−0.30	6.82	1.12
c33810_g2	UDP-glycosyltransferase 74E1-like (UGT74E1)	0.93	−1.09	2.01	0.87	1.65	0.22
c37821_g2	UDP-glycosyltransferase 74E1-like (UGT74E1)	1.56	−1.00	2.86	−0.01	4.18	1.00
c34021_g2	UDP-glycosyltransferase 74F2-like (UGT74F2)	6.67	−1.14	9.71	0.44	10.21	0.70
c33043_g1	UDP-glycosyltransferase 74F2-like (UGT74F2)	0.82	−0.67	1.71	1.15	0.91	−0.48
c30945_g2	UDP-glycosyltransferase 76F1-like (UGT76F1)	0.84	−1.15	2.02	0.56	2.04	0.59
c38976_g2	UDP-glycosyltransferase 79B3-like (UGT79B3)	0.64	−0.88	3.37	1.09	1.56	−0.21
c31051_g1	UDP-glycosyltransferase 82A1-like (UGT82A1)	8.77	0.29	9.52	0.82	6.77	−1.11
c28250_g1	UDP-glycosyltransferase 85A1-like (UGT85A1)	94.13	−0.66	97.56	−0.49	128.87	1.15
c24625_g2	UDP-glycosyltransferase 85A2-like (UGT85A2)	7.41	−0.61	9.28	1.15	7.48	−0.54
c40172_g1	UDP-glycosyltransferase 85A2-like (UGT85A2)	194.98	0.18	201.03	0.90	184.47	−1.08
c34336_g2	UDP-glycosyltransferase 85A2-like (UGT85A2)	3.71	−0.83	5.16	−0.28	8.81	1.11
c35036_g2	UDP-glycosyltransferase 85A2-like, partial (UGT85A2)	0.44	−1.03	4.26	0.97	2.54	0.07
c35036_g3	UDP-glycosyltransferase 85A3-like (UGT85A3)	15.82	−0.69	18.69	1.15	16.2	−0.45
c35036_g8	UDP-glycosyltransferase 85A3-like (UGT85A3)	28.3	−0.06	32.03	1.03	25.21	−0.97
c61574_g1	UDP-glycosyltransferase 85A3-like (UGT85A3)	1.16	−0.99	3.49	1.01	2.29	−0.02
c40692_g4	UDP-glycosyltransferase 85A3-like (UGT85A3)	7.59	−1.01	10.75	0.99	9.23	0.03
c24625_g1	UDP-glycosyltransferase 85A5-like (UGT85A5)	5.43	−0.35	7.22	1.13	4.91	−0.78
c41014_g1	UDP-glycosyltransferase 86A1-like (UGT86A1)	28.94	−1.12	35.75	0.33	37.95	0.80
c27184_g1	hexokinase-1-like (HXK1)	12.24	−1.15	14.26	0.69	14.00	0.45
c27184_g2	hexokinase-1-like (HXK1)	12.15	−0.69	13.01	1.15	12.26	−0.46
c37571_g2	hexokinase-1-like (HXK1)	86.71	0.96	82.88	−1.03	85.00	0.07
c35355_g1	hexokinase-2, chloroplastic-like (HXK2)	22.68	1.05	19.37	−0.94	20.77	−0.10
**Starch biosynthetic and metabolic process**							
c33953_g1	granule-bound starch synthase 1, chloroplastic/amyloplastic-like (GBSS1)	31.55	−0.86	55.03	1.10	38.98	−0.24
c32146_g1	starch synthase 1, chloroplastic/amyloplastic-like (SS1)	7.33	−1.14	9.39	0.73	9.04	0.41
c36949_g1	starch synthase 2, chloroplastic/amyloplastic-like (SS2)	18.01	−0.48	21.13	1.15	17.63	−0.67
c38296_g1	soluble starch synthase 3, chloroplastic/amyloplastic-like (SS3)	10.30	−1.04	13.61	0.95	12.19	0.09
c37526_g1	probable starch synthase 4, chloroplastic/amyloplastic-like (SS4)	14.16	−0.86	16.81	1.10	15.00	−0.24
c71775_g1	thiazole biosynthetic enzyme (THI1)	0.00	−0.58	0.00	−0.58	1.12	1.15
c35594_g1	sedoheptulose-1,7-bisphosphatase, chloroplastic-like (SBPase)	66.93	−1.12	119.29	0.79	106.87	0.34
c53767_g1	starch phosphorylase 3 (SP3)	0.65	1.15	0.36	−0.49	0.33	−0.66
c35552_g1	ADP-glucose pyrophosphorylase small subunit (APS)	30.58	−1.11	33.91	0.29	35.15	0.82
c32549_g2	probable alpha-amylase 2-like (AMY2)	14.78	−1.05	17.36	0.94	16.30	0.12
c37246_g3	alpha-glucan phosphorylase, H isozyme-like (BAM)	37.12	−0.73	38.83	−0.41	47.11	1.14
c34599_g1	beta-amylase 1 (BAM1)	24.97	−0.86	39.67	1.10	29.57	−0.24
c39970_g1	beta-amylase 3, chloroplastic-like (BAM3)	22.39	−1.03	29.89	0.06	36.12	0.97
c32387_g1	beta-amylase 3, chloroplastic-like (BAM3)	9.43	−0.61	10.36	1.15	9.47	−0.54
c37854_g3	beta-amylase 7-like (BAM7)	17.01	−0.34	18.51	1.13	16.56	−0.78
c31912_g1	inactive beta-amylase 9-like [Fragaria vesca subsp. vesca] (IBAM9)	42.29	0.28	47.57	0.83	28.85	−1.11
**Sugar transport**							
c38802_g4	sucrose proton symporter 2 (SUC2)	28.45	−0.45	5.18	1.15	3.69	−0.70
c28789_g1	sucrose transport protein SUC2-like (SUC2)	0	−0.09	25.95	−0.95	13.22	1.04
c31134_g2	sucrose transport protein SUC3-like isoform 2 (SUT3)	16.42	0.97	145.21	−1.03	138.62	0.06
c28824_g2	sucrose transport protein SUC3-like isoform 2 (SUT3)	19.07	−0.25	16.66	1.10	19.97	−0.85
c34816_g3	sucrose transport protein SUC3-like isoform 2 (SUT3)	14.93	−0.77	27.06	1.13	31.46	−0.36
c34147_g1	sugar transport protein 13-like (SUT13)	15.33	−1.10	24.29	0.24	25.05	0.86
c22780_g1	UDP-sugar transporter UST74c-like (UST74)	0.29	−0.18	0.3	1.08	0.3	−0.90
c24164_g1	UDP-sugar transporter UST74c-like (UST74)	0.54	0.87	0	−1.09	0.44	0.22
c34518_g1	bidirectional sugar transporter NEC1-like (NEC1)	3.29	−1.08	2.97	0.19	2.45	0.89
c52229_g1	LOW QUALITY PROTEIN: sugar transporter ERD6-like (ERD6)	0.25	−1.07	0.45	0.92	0.2	0.15
c40766_g1	Nucleotide-sugar transporter family protein (NSTP)	23.54	0.31	18.51	0.81	18.12	−1.12
c32423_g4	putative UDP-sugar transporter DDB_G0278631-like (UST)	2.61	−0.28	0.94	−0.83	1.01	1.11
c28360_g1	major facilitator superfamily domain-containing protein 5-like (MSS5)	3.99	0.07	18.75	0.96	19.88	−1.03
c31443_g1	major facilitator superfamily domain-containing protein 5-like (MSS5)	18.41	0.09	41.97	−1.04	56	0.95
c31998_g1	major facilitator superfamily domain-containing protein 5-like (MSS5)	0.97	−0.24	6.25	−0.86	7.31	1.10
c38009_g1	major facilitator superfamily domain-containing protein 12-like (MSS12)	142.27	−1.00	0.51	−0.00	0.53	1.00
c38009_g3	major facilitator superfamily domain-containing protein 12-like (MSS12)	18.55	−1.15	4.61	0.58	2.82	0.58
c60302_g1	senescence-associated protein SAG21 (SAG21)	0	−0.58	13.88	−0.58	14.67	1.15
**Glucose biosynthetic and metabolic process**							
c27051_g1	alpha,alpha-trehalose-phosphate synthase [UDP-forming] 1-like (TPS1)	0.62	−0.61	0.64	−0.54	1.17	1.15
c39389_g1	alpha,alpha-trehalose-phosphate synthase [UDP-forming] 1-like (TPS1)	23.85	−0.85	24.31	−0.26	25.38	1.10
c28659_g1	alpha,alpha-trehalose-phosphate synthase [UDP-forming] 1-like (TPS1)	0.73	−0.86	0.87	−0.24	1.17	1.10
c36251_g3	alpha,alpha-trehalose-phosphate synthase [UDP-forming] 5-like (TPS5)	42.41	0.55	38.55	−1.15	42.55	0.61
c24788_g1	alpha,alpha-trehalose-phosphate synthase [UDP-forming] 6-like (TPS6)	33.03	0.42	34.53	0.72	25.17	−1.14
c32576_g1	probable alpha,alpha-trehalose-phosphate synthase [UDP-forming] 7 (TPS7)	46.77	−0.23	56.18	1.09	42.21	−0.87
c23560_g1	probable alpha,alpha-trehalose-phosphate synthase [UDP-forming] 9-like (TPS9)	3.02	−0.27	3.96	1.11	2.64	−0.83
c23603_g1	probable alpha,alpha-trehalose-phosphate synthase [UDP-forming] 9-like (TPS9)	2.34	−0.39	3.33	1.14	2.11	−0.75
c27119_g1	probable alpha,alpha-trehalose-phosphate synthase [UDP-forming] 9-like (TPS9)	5.91	−0.34	8.42	1.13	5.14	−0.79
c37734_g2	probable alpha,alpha-trehalose-phosphate synthase [UDP-forming] 10-like (TPS10)	34.51	0.40	36.24	0.74	26.71	−1.14
c36419_g1	probable alpha,alpha-trehalose-phosphate synthase [UDP-forming] 11-like (TPS11)	2.2	1.04	1.59	−0.09	1.12	−0.95
c36419_g4	probable alpha,alpha-trehalose-phosphate synthase [UDP-forming] 11-like (TPS11)	2.41	1.04	1.8	−0.08	1.32	−0.96
c30694_g1	trehalose-phosphate phosphatase A-like (TPP)	15.34	0.09	17.95	0.95	11.87	−1.04
c29265_g1	probable trehalose-phosphate phosphatase D-like (TPP)	3.5	0.78	3.29	0.34	2.59	−1.13
c30405_g1	probable trehalose-phosphate phosphatase F-like (TPP)	3.51	−0.36	4.93	1.13	3.11	−0.77
c26335_g1	probable trehalose-phosphate phosphatase J (TPP)	23.89	0.67	23.08	0.48	16.1	−1.15
c20081_g1	probable trehalose-phosphate phosphatase J-like (TPP)	17.92	0.74	17.03	0.40	13.06	−1.14
c31619_g1	probable trehalose-phosphate phosphatase 4 (TPP4)	0.53	0.38	0.54	0.76	0.49	−1.13
c65406_g1	glyceraldehyde-3-phosphate dehydrogenase A subunit (GAPA)	0	−0.58	0	−0.58	1.57	1.15
c25086_g1	glyceraldehyde-3-phosphate dehydrogenase A, chloroplastic-like (GAPA)	417.54	−1.15	599	0.66	581.45	0.49
c33989_g1	glyceraldehyde-3-phosphate dehydrogenase, cytosolic-like (GAPA)	88.17	0.81	83.86	−1.12	87.03	0.30
c35001_g3	glyceraldehyde-3-phosphate dehydrogenase-like isoform X1 (GAPA)	0	−0.58	0.84	1.15	0	−0.58
c33013_g1	glycolytic glyceraldehyde-3-phosphate dehydrogenase (GAPA)	0.64	−0.43	4.02	1.14	0.05	−0.71
c20128_g1	putative glyceraldehyde-3-phosphate dehydrogenase (GAPA)	0	−0.58	0	−0.58	0.8	1.15
c48240_g1	Glyceraldehyde-3-phosphate dehydrogenase A, chloroplastic (GAPA)	0	−0.58	0	−0.58	3.11	1.15
c66389_g1	glyceraldehyde 3-phosphate dehydrogenase GAPA2 (GAPA2)	0	−0.58	0	−0.58	0.56	1.15
c73506_g1	glyceraldehyde 3-phosphate dehydrogenase GAPA2 (GAPA2)	0	−0.58	0	−0.58	0.47	1.15
c9107_g1	glyceraldehyde-3-phosphate dehydrogenase B (GAPB)	0	−0.58	0	−0.58	1.07	1.15
c31412_g1	glyceraldehyde-3-phosphate dehydrogenase B, chloroplastic-like (GAPB)	52.04	−1.06	83.08	0.13	103.79	0.93
c39734_g4	glyceraldehyde-3-phosphate dehydrogenase GAPCP1, chloroplastic-like (GAPCP1)	11.25	−0.86	12.31	−0.24	14.58	1.10
