# Supplementary material for: Transcriptome of the floral transition in Rosa chinensis ‘Old Blush’
Source: BMC Genomics. 2017 Feb 23;18:199. doi: 10.1186/s12864-017-3584-y (PMC5322666; doi:10.1186/s12864-017-3584-y)
Supplement: Additional file 14: — Selection of hormones - related differentially expressed genes in rose. (PDF 535 kb) [file 12864_2017_3584_MOESM14_ESM.pdf]

Additional file 14 Selection of hormone - related differentially expressed genes in rose

Rose genes Identification	Annotation	VM FPKM	VM Z-score (FPKM)	TM FPKM	TM Z-score (FPKM)	FM FPKM	FM Z-score (FPKM)
**GA related**							
c18828_g1	ent-copalyl diphosphate synthase, chloroplastic-like (CPS)	0.21	–0.80	0.35	–0.31	0.76	1.12
c35978_g1	ent-copalyl diphosphate synthase, chloroplastic-like (CPS)	7.81	0.00	7.36	–1.00	8.26	1.00
c42073_g1	ent-kaurene synthase (KS)	0.31	0.06	0.57	0.97	0	–1.03
c32128_g1	ent-kaurene oxidase, chloroplastic-like (KO)	6.21	–1.04	6.89	0.09	7.41	0.95
c46612_g1	putative cytochrome P450 monooxygenase (CYPM)	0	–0.58	0	–0.58	0.37	1.15
c24362_g1	gibberellin 20 oxidase 1-like (GA20ox1)	10.33	0.89	7.84	–1.08	9.45	0.19
c32329_g1	gibberellin 20 oxidase 1-like (GA20ox1)	3.41	1.06	2.88	–0.14	2.53	–0.93
c36047_g2	gibberellin 20 oxidase 1-like (GA20ox1)	1.26	–0.01	1.49	1.01	1.04	–0.99
c13378_g1	gibberellin 20 oxidase 1-like (GA20ox1)	2.98	–0.56	4.93	1.15	2.95	–0.59
c25609_g2	gibberellin 20 oxidase 1-like (GA20ox1)	3.52	–0.77	3.76	–0.36	4.61	1.13
c32329_g2	gibberellin 20 oxidase 1-like (GA20ox1)	2.39	1.15	1.42	–0.55	1.39	–0.60
c30627_g1	gibberellin 20 oxidase 1-like (GA20ox1)	0.92	–0.14	0.74	–0.92	1.2	1.06
c71469_g1	gibberellin 2-beta-dioxygenase 2-like (GA2ox2)	0.26	0.35	0.28	0.78	0.19	–1.13
c46405_g1	gibberellin 2-beta-dioxygenase 8-like (GA2ox8)	0	–0.58	2.23	1.15	0	–0.58
c39921_g2	gibberellin 2-beta-dioxygenase 8-like (GA2ox8)	25.96	–0.97	30.49	1.03	28.04	–0.05
c60372_g1	gibberellin 3-beta-dioxygenase 1-like (GA3ox1)	0.07	1.15	0.06	–0.58	0.06	–0.58
c34390_g1	gibberellin 3-beta-dioxygenase 3-like (GA3ox3)	19.79	0.82	16.9	–1.11	19	0.29
c23089_g1	gibberellin 3-beta-dioxygenase 3-like (GA3ox3)	0.27	–0.58	0.27	–0.58	0.54	1.15
c23421_g1	gibberellin 3-oxidase (GA3ox)	1.75	0.44	1.81	0.71	1.4	–1.14
c29640_g2	gibberellin receptor GID1B-like (GID1B)	25.55	0.72	24.87	0.42	21.36	–1.14
c28645_g1	F-box protein GID2-like (GID2)	68.93	–0.60	72.12	1.15	69.03	–0.55
c30274_g1	putative SPINDLY protein (SPY)	28.28	0.38	28.85	0.76	25.99	–1.13
c32788_g1	SEC1 family transport protein SLY1-like (SLY1)	35.29	–0.57	37.29	1.15	35.27	–0.59
c37090_g1	DELLA protein GAI-like (GAI)	32.44	–0.88	34.05	–0.21	37.14	1.09
c62836_g1	DELLA protein GAI-like (GAI)	0	–0.58	0.32	1.15	0	–0.58
c74431_g1	DELLA protein GAI-like (GAI)	0	–0.58	1.21	1.15	0	–0.58
c40167_g1	GAI1	54.53	0.73	52.99	–1.14	54.26	0.41
c33244_g1	DELLA protein GAI1-like (GAI1)	21.12	–0.86	24.66	1.10	22.25	–0.24
c11240_g1	DELLA protein RGL1-like (RGL)	0.19	1.11	0.11	–0.29	0.08	–0.82
c48118_g1	DELLA protein RGL1-like (RGL)	0.13	–0.43	0.24	1.14	0.11	–0.71
c34645_g1	scarecrow-like protein 4-like (SCL4)	18.51	1.15	16.45	–0.52	16.32	–0.63
c6885_g1	scarecrow-like protein 13-like (SCL13)	58.56	0.75	33.04	–1.14	53.71	0.39
c20369_g1	scarecrow-like protein 13-like (SCL13)	55.84	–0.46	34.35	1.15	53.32	–0.68
c37717_g1	scarecrow-like protein 15-like (SCL15)	11.4	–0.97	14.13	–0.05	17.29	1.02
c30880_g1	scarecrow-like protein 32-like (SCL32)	3.67	1.15	2.71	–0.52	2.65	–0.63
c32681_g1	scarecrow-like protein 32-like (SCL32)	6.68	–0.67	8.62	1.15	6.89	–0.48
c33112_g2	scarecrow-like transcription factor PAT1-like (PAT1)	10.86	0.84	10	–1.11	10.61	0.27
c31121_g2	transcription factor GAMYB-like (GAMYB)	6.35	–0.22	7.94	1.09	5.55	–0.87
c23325_g1	gibberellin-regulated protein 6-like (GASA6)	176.92	0.84	116.91	–1.11	159.57	0.27
c27831_g1	gibberellin-regulated protein 11-like (GASA11)	17.78	0.67	13.86	–1.15	17.35	0.47
c15202_g1	Myb domain protein 27, putative (MYB27)	0.26	0.53	0.08	–1.15	0.27	0.62
c71266_g1	Myb domain protein 103 (MYB103)	0.11	–0.16	0.29	1.07	0	–0.91
**IAA related**							
c40925_g1	probable flavin-containing monooxygenase 1-like (YUC1)	6.95	–1.07	11.40	0.15	14.21	0.92
c28926_g1	probable flavin-containing monooxygenase 1-like (YUC1)	17.05	–0.62	21.16	1.15	17.25	–0.53
c33262_g2	flavin-containing monooxygenase FMO GS-OX-like 3-like (FMO GS-OX3)	5.71	–0.86	6.51	1.10	5.96	–0.24
c36574_g2	auxin transporter-like protein 2-like (LAX2)	80.85	1.12	68.53	–0.81	71.68	–0.31
c14885_g1	auxin transporter-like protein 2-like (LAX2)	45.62	–1.12	48.52	0.32	49.50	0.80
c25158_g1	auxin transporter-like protein 2-like (LAX2)	18.29	–0.98	22.02	–0.04	26.22	1.02
c28247_g1	auxin transporter-like protein 3-like (LAX3)	20.69	–0.88	22.32	–0.21	25.47	1.09
c36372_g2	auxin-induced protein 10A5-like (AUX10A5)	5.40	0.71	3.05	–1.14	5.05	0.43
c24077_g1	auxin-induced protein AUX22-like (AUX22)	12.33	–0.94	17.96	–0.11	25.74	1.05
c34161_g2	auxin-induced protein AUX28-like (AUX28)	109.60	–0.86	118.50	–0.24	137.50	1.10
c26497_g1	auxin-responsive protein IAA4-like (IAA4)	19.02	–1.15	35.60	0.63	34.61	0.52
c10725_g1	auxin-responsive protein IAA14-like (IAA14)	49.98	–0.92	63.15	1.06	55.18	–0.14
c27691_g1	auxin-responsive protein IAA17-like (IAA17)	46.76	–1.06	53.89	0.93	51.03	0.13
c38439_g1	auxin-responsive protein IAA27-like (IAA27)	136.23	–0.77	147.51	1.13	138.63	–0.36
c40768_g1	auxin-induced protein 5NG4-like (5NG4)	18.04	0.54	12.97	–1.15	18.25	0.61
c39136_g2	auxin-induced protein 5NG4-like (5NG4)	135.13	1.07	112.08	–0.91	120.79	–0.16
c38861_g1	indole-3-acetic acid-induced protein ARG7-like (SAUR)	225.17	0.97	161.13	–1.03	196.00	0.06
c34702_g1	probable indole-3-acetic acid-amido synthetase GH3.5-like (GH3.5)	19.71	–1.15	22.28	0.68	21.97	0.46
c30709_g1	probable indole-3-acetic acid-amido synthetase GH3.5-like (GH3.5)	33.65	0.46	34.32	0.69	28.91	–1.15
c31167_g1	indole-3-acetic acid-amido synthetase GH3.6-like (GH3.6)	37.00	0.99	28.79	0.01	20.32	–1.01
c37665_g1	auxin efflux carrier (PIN)	61.05	1.10	57.07	–0.25	55.28	–0.85
c33533_g1	probable auxin efflux carrier component 1b-like (PIN1b)	9.79	1.11	7.68	–0.28	6.83	–0.83
c33704_g1	auxin efflux carrier component 3-like (PIN3)	46.38	–0.78	50.70	1.13	47.35	–0.35
c33704_g3	auxin efflux carrier component 3-like (PIN3)	32.14	–1.01	42.88	0.98	37.75	0.03
c28000_g1	auxin-binding protein ABP19a-like (ABP19a)	211.60	–0.96	293.30	–0.07	395.67	1.04
c65049_g1	auxin-binding protein ABP19a-like (ABP19a)	176.30	–0.83	235.12	–0.28	386.21	1.11
c25446_g1	auxin-binding protein ABP19a-like (ABP19a)	18.25	–0.87	26.37	–0.22	42.53	1.09
c28000_g2	auxin-binding protein ABP20 (ABP20)	58.60	–0.89	95.67	–0.20	164.09	1.08
**ABA related**							
c30773_g1	9-cis-epoxycarotenoid dioxygenase, chloroplastic-like (NCED1)	27.24	0.58	14.92	–1.15	27.25	0.58
c19935_g1	abscisic acid receptor PYR1-like (PYR1)	8.84	1.15	4.55	–0.69	5.09	–0.46
c25470_g1	abscisic acid receptor PYL4-like (PYL4)	48.72	1.04	37.26	–0.09	28.62	–0.95
c31817_g5	abscisic acid receptor PYL6-like (PYL6)	35.86	1.05	22.81	–0.94	28.19	–0.12
c28743_g1	abscisic acid receptor PYL8-like (PYL8)	35.56	1.14	28	–0.74	29.4	–0.39
c36777_g7	abscisic acid receptor PYL9-like (PYL9)	4.28	1.09	2.4	–0.22	1.47	–0.87
c32685_g1	ammonium transporter 3 member 1-like (AMT3)	2.42	–1.05	5.13	0.10	7.1	0.94
c33120_g1	bZIP transcription factor 60-like (bZIP60)	92.57	1.07	55.32	–0.92	69.8	–0.15
c31110_g1	probable protein phosphatase 2C 26-like (PP2C26)	9.69	–1.14	11.54	0.44	11.86	0.71
c31589_g2	probable protein phosphatase 2C 33-like (PP2C33)	27.12	–0.88	30.51	1.09	28.29	–0.20
c33570_g1	probable protein phosphatase 2C 33-like (PP2C33)	17.54	1.12	14.6	–0.82	15.39	–0.30
c39671_g2	probable protein phosphatase 2C 34-like (PP2C34)	71.04	–0.44	74.48	1.15	70.48	–0.70
c34941_g2	protein phosphatase 2C 37-like (PP2C37)	56.14	0.14	58.1	0.92	53.09	–1.06
c19598_g1	probable protein phosphatase 2C 38-like (PP2C38)	7.31	–0.26	8.71	1.10	6.71	–0.84
c30794_g2	probable protein phosphatase 2C 40-like isoform 1 (PP2C40)	9.34	–0.94	10.07	–0.10	11.07	1.05
c35618_g1	probable protein phosphatase 2C 55-like (PP2C55)	64.9	–0.71	74.03	1.14	66.31	–0.43
c39010_g1	protein phosphatase 2C 57-like (PP2C57)	11.33	–1.15	18.44	0.67	17.72	0.48
c33651_g1	probable protein phosphatase 2C 59-like (PP2C59)	66.15	–0.75	71.98	1.14	67.25	–0.39
c33584_g1	probable protein phosphatase 2C 60-like (PP2C60)	102.11	1.07	96.24	–0.15	92.58	–0.91
c35070_g1	probable protein phosphatase 2C 76-like (PP2C76)	54.05	–1.12	59.34	0.30	61.28	0.82
c32996_g2	protein phosphatase 2C 77 (PP2C77)	4.77	–0.81	6.11	–0.31	9.9	1.12
c28268_g2	SNF1-related protein kinase catalytic subunit alpha KIN10-like (KIN10)	49.96	1.03	47.57	–0.96	44.48	–0.07
c37976_g1	SNF1-related protein kinase regulatory subunit gamma-1-like (KING1/SnRK1)	12.75	0.94	10.43	0.12	7.11	–1.05
c36302_g3	transcription factor MYC2 (MYC2)	98.69	0.79	57.82	–1.12	88.88	0.33
c40347_g1	transcription factor MYC2-like (MYC2)	98.9	0.82	77.98	–1.11	93.12	0.29
