# Supplementary material for: Transcriptome of the floral transition in Rosa chinensis ‘Old Blush’
Source: BMC Genomics. 2017 Feb 23;18:199. doi: 10.1186/s12864-017-3584-y (PMC5322666; doi:10.1186/s12864-017-3584-y)
Supplement: Additional file 15: — Selection flower - related differentially expressed genes in rose. (PDF 725 kb) [file 12864_2017_3584_MOESM15_ESM.pdf]

Additional file 15 Selection flower - related differentially expressed genes in rose

Rose genes Identification	Annotation	VM		TM		FM	
		FPKM	Z-score (FPKM)	FPKM	Z-score (FPKM)	FPKM	Z-score (FPKM)
**Flower integrator**							
c19624_g1	flowering locus T protein (FT)	0.49	1.08	0.31	−0.19	0.21	−0.89
c34960_g1	SOC1-like protein (SOC1)	81.16	−0.98	87.56	−0.04	94.86	1.02
c40205_g1	MADS-box protein SOC1-like (SOC1)	21.76	−0.85	22.78	−0.25	25.1	1.10
c28547_g1	LEAFY protein (LFY)	2.93	1.15	1.53	−0.49	1.38	−0.66
c23927_g1	APETALA1-like protein (AP1)	1.25	−0.84	1.77	−0.27	3.01	1.11
c22434_g1	putative APETALA1 protein (AP1)	7.66	−0.96	10.24	−0.08	13.54	1.04
c28239_g1	APETALA1/FUL-like protein (AP1)	3.35	−1.15	5.16	0.56	5.19	0.59
c28628_g1	CONSTANS (CO)	10.02	−0.10	18.06	1.05	4.05	−0.95
c25884_g1	protein FD-like (FD)	10.21	1.14	9.29	−0.70	9.42	−0.44
c36047_g1	protein FD-like (FD)	2	1.08	1.6	−0.18	1.37	−0.90
c28601_g1	protein FD-like (FD)	4.51	−1.14	5.08	0.42	5.19	0.72
c2426_g1	PEBP	0.21	1.02	0.14	−0.05	0.08	−0.97
c48729_g1	PEBP-like protein (PEBP)	0.25	−0.27	0.85	1.11	0	−0.84
c16305_g1	terminal flower 1 (TFL1)	1.02	−0.96	1.39	−0.08	1.86	1.04
**Flower development**							
c53833_g1	floral homeotic protein AGAMOUS-like isoform X2 (AG)	0.00	−0.58	0.49	1.15	0.00	−0.58
c33337_g1	agamous-like MADS-box protein AGL8-like (AGL8)	0.86	−1.15	1.41	0.49	1.47	0.66
c21152_g1	agamous-like MADS-box protein AGL11-like isoform X3 (AGL11)	0.00	−0.58	0.78	1.15	0.00	−0.58
c12965_g1	agamous-like MADS-box protein AGL12-like (AGL12)	0.22	−1.14	0.33	0.43	0.35	0.71
c34686_g1	agamous-like MADS-box protein AGL19-like (AGL19)	45.60	−0.98	49.39	1.02	47.37	−0.04
c26775_g2	agamous-like MADS-box protein AGL61-like (AGL61)	0.21	−0.47	0.20	−0.68	0.29	1.15
c6863_g1	agamous-like MADS-box protein AGL62-like (AGL62)	0.27	−1.15	0.49	0.46	0.52	0.68
c26190_g1	developmental protein SEPALLATA 2-like (SEP2)	0.86	−1.10	1.52	0.85	1.32	0.26
c27470_g1	MADS transcriptional factor SEP3 homolog, partial (SEP3)	0.26	−1.09	0.70	0.88	0.55	0.21
c40420_g5	MADS6	34.44	−0.59	34.56	−0.57	46.20	1.15
c11711_g1	MADS-box	0.00	−0.58	0.69	1.15	0.00	−0.58
c26338_g2	MADS-box protein (MADS-box)	0.04	−0.98	0.21	1.02	0.12	−0.04
c8807_g1	MADS-box protein (MADS-box)	0.12	−0.17	0.05	−0.90	0.24	1.08
c22472_g1	MADS-box protein (MADS-box)	0.77	−0.96	1.34	−0.08	2.07	1.04
c26338_g1	MADS-box protein (MADS-box)	4.52	−1.15	6.90	0.56	6.94	0.59
c14342_g1	MADS-box transcription factor 6-like (MADS-box6)	0.10	−1.07	0.31	0.91	0.23	0.16
c44156_g1	MADS-box transcription factor 17-like (MADS-box17)	0.94	−0.07	1.56	1.03	0.44	−0.96
c65440_g1	MADS-box transcription factor 17-like (MADS-box17)	0.10	−0.77	0.24	1.13	0.13	−0.36
c18612_g1	MADS-box protein CMB1-like (CMB1)	0.28	−0.55	0.27	−0.60	0.60	1.15
c36317_g2	MADS-box protein SVP-like (SVP)	5.01	−1.11	6.74	0.26	7.47	0.84
c37522_g1	MADS-box protein SVP-like (SVP)	44.94	0.31	47.81	0.81	36.60	−1.12
c38302_g2	putative EMF1 protein (EMF1)	5.68	0.23	4.86	−1.10	6.07	0.86
c34647_g1	polycomb group protein EMBRYONIC FLOWER 2-like (EMF2)	10.24	−0.91	11.01	1.07	10.53	−0.16
c35736_g2	polycomb group protein EMBRYONIC FLOWER 2-like (EMF2)	38.93	0.83	37.10	−1.11	38.42	0.29
c27026_g1	NAC domain-containing protein 2-like (NAC2)	35.48	−1.06	43.58	0.14	48.87	0.92
c27026_g2	NAC domain-containing protein 2-like (NAC2)	47.28	−0.29	42.23	−0.82	60.54	1.11
c25248_g2	NAC domain-containing protein 7-like (NAC7)	3.20	−1.05	3.53	0.12	3.76	0.94
c26738_g1	NAC domain-containing protein 7-like (NAC7)	0.60	−1.14	0.99	0.43	1.06	0.71
c65531_g1	NAC domain-containing protein 7-like (NAC7)	0.21	0.02	0.41	0.99	0.00	−1.01
c26738_g2	NAC domain-containing protein 7-like (NAC7)	1.44	−1.14	1.87	0.41	1.96	0.73
c20155_g1	NAC domain-containing protein 8-like (NAC8)	0.54	−1.06	1.40	0.92	1.06	0.14
c30958_g1	NAC domain-containing protein 8-like (NAC8)	28.30	0.05	28.77	0.98	27.76	−1.02
c31181_g1	NAC domain-containing protein 8-like (NAC8)	1.11	−1.11	1.71	0.28	1.95	0.83
c27463_g1	NAC domain-containing protein 8-like (NAC8)	1.00	0.43	1.02	0.71	0.89	−1.14
c28429_g1	NAC domain-containing protein 8-like isoform 1 (NAC8)	2.87	−1.15	4.82	0.69	4.57	0.46
c11316_g1	NAC transcription factor 25-like (NAC25)	0.76	−1.14	1.08	0.74	1.02	0.39
c22647_g1	NAC transcription factor 29-like (NAC29)	0.67	−0.92	2.04	1.06	1.21	−0.14
c22647_g2	NAC transcription factor 29-like (NAC29)	2.58	−0.07	2.91	1.03	2.31	−0.97
c39264_g1	NAC transcription factor 29-like (NAC29)	4.75	0.01	5.10	1.00	4.39	−1.00
c14811_g1	NAC domain-containing protein 43-like (NAC43)	0.26	−0.34	0.22	−0.79	0.39	1.13
c15398_g1	NAC domain-containing protein 43-like (NAC43)	0.17	−1.09	0.32	0.87	0.27	0.22
c29089_g1	NAC domain-containing protein 43-like (NAC43)	5.44	0.06	5.63	0.97	5.21	−1.03
c40384_g2	NAC domain-containing protein 43-like (NAC43)	4.23	−0.91	4.38	−0.16	4.63	1.07
c40246_g2	NAC domain-containing protein 48-like (NAC48)	4.88	−1.03	7.08	0.07	8.85	0.96
c33208_g1	NAC domain-containing protein 72-like (NAC72)	17.92	−0.86	23.25	−0.23	34.49	1.10
c39642_g5	NAC domain-containing protein 72-like (NAC72)	8.58	0.33	8.69	0.79	8.23	−1.12
c30635_g1	NAC domain-containing protein 74-like (NAC74)	2.66	1.15	0.37	−0.44	0.00	−0.70
c36239_g2	NAC domain-containing protein 74-like (NAC74)	0.90	−0.22	1.79	1.09	0.46	−0.87
c36239_g3	NAC domain-containing protein 74-like (NAC74)	1.87	1.12	0.85	−0.31	0.49	−0.81
c40933_g1	NAC domain-containing protein 74-like (NAC74)	5.99	0.25	7.18	0.85	3.29	−1.10
c25637_g1	NAC domain-containing protein 78-like (NAC78)	1.84	−0.64	1.86	−0.51	2.13	1.15
c29211_g2	NAC domain-containing protein 78-like (NAC78)	6.05	−0.89	7.11	−0.20	9.07	1.08
c29959_g1	NAC domain-containing protein 78-like (NAC78)	67.15	−0.80	69.12	1.12	67.64	−0.32
c34481_g1	NAC domain-containing protein 89-like (NAC89)	5.74	0.30	5.81	0.82	5.55	−1.11
c16109_g1	NAC domain-containing protein 90-like (NAC90)	14.86	−0.45	13.68	−0.70	22.32	1.15
c335_g1	NAC domain-containing protein 90-like (NAC90)	0.56	−0.45	0.49	−0.70	1.00	1.15
c21455_g1	NAC domain protein 100 (NAC100)	0.31	−0.68	0.36	−0.46	0.73	1.15
c38951_g3	NAC domain-containing protein 100-like isoform 1 (NAC100)	1.61	−0.75	1.72	−0.39	2.19	1.14
c27568_g1	NAC transcription factor ONAC010-like (0NAC010)	3.09	0.22	3.31	0.87	2.65	−1.09
c38399_g2	squamosa promoter-binding protein 1-like (SPL1)	56.01	−0.92	64.30	−0.15	77.56	1.07
c16905_g1	squamosa promoter-binding protein 1-like (SPL1)	22.73	1.08	17.29	−0.90	19.29	−0.17
c28584_g1	squamosa promoter-binding-like protein 1-like (SPL1)	32.01	−0.88	33.97	−0.20	37.66	1.09
c28584_g2	squamosa promoter-binding-like protein 1-like (SPL1)	40.47	−0.81	41.05	−0.31	42.74	1.12
c31417_g1	squamosa promoter-binding-like protein 4-like (SPL4)	5.62	−1.15	7.78	0.53	7.89	0.62
c26494_g2	squamosa promoter-binding-like protein 6-like (SPL6)	3.36	−1.11	3.98	0.30	4.21	0.82
c10634_g1	squamosa promoter-binding-like protein 7-like (SPL7)	3.10	0.77	2.78	0.35	1.65	−1.13
c30356_g3	squamosa promoter-binding-like protein 7-like (SPL7)	0.23	−0.80	0.28	−0.32	0.43	1.12
c39234_g1	squamosa promoter-binding-like protein 7-like (SPL7)	22.88	0.94	22.60	0.11	22.21	−1.05
c34523_g1	squamosa promoter-binding-like protein 8-like (SPL8)	30.81	1.12	26.33	−0.79	27.40	−0.33
c39698_g2	squamosa promoter-binding-like protein 12-like (SPL12)	66.16	−1.14	67.60	0.43	67.86	0.71
c26815_g1	squamosa promoter-binding-like protein 13A-like (SPL13)	19.58	−1.15	25.56	0.55	25.73	0.60
c36772_g2	squamosa promoter-binding-like protein 13A-like (SPL13)	23.28	−0.99	28.56	1.01	25.86	−0.02
c40535_g2	squamosa promoter-binding-like protein 14-like (SPL14)	34.35	−1.14	38.42	0.70	37.84	0.44
c33083_g3	squamosa promoter-binding-like protein 17-like (SPL17)	33.49	−0.77	34.52	1.13	33.71	−0.36
c21416_g1	transcription factor TCP4-like (TCP4)	11.18	1.06	10.07	−0.93	10.52	−0.13
c40872_g1	transcription factor TCP7-like (TCP7)	74.34	1.12	65.11	−0.81	67.53	−0.31
c22464_g1	transcription factor TCP9-like (TCP9)	10.80	−0.10	12.58	1.05	9.48	−0.95
c26666_g1	transcription factor TCP9-like (TCP9)	19.91	0.79	19.20	0.33	16.94	−1.12
c39202_g3	transcription factor TCP9-like (TCP9)	26.62	0.37	28.87	0.76	17.95	−1.13
c30691_g1	transcription factor TCP13-like (TCP13)	12.56	0.65	12.35	0.50	9.90	−1.15
c33599_g4	transcription factor TCP14-like (TCP14)	27.60	−0.70	31.95	1.14	28.22	−0.44
c20750_g1	transcription factor TCP14-like isoform 1 (TCP14)	22.38	−0.82	23.33	1.11	22.64	−0.29
c28771_g1	transcription factor TCP19-like (TCP19)	6.29	0.71	5.89	−1.14	6.23	0.43
c27987_g3	probable mediator of RNA polymerase II transcription subunit 37c-like (CCT)	89.77	1.00	54.00	0.01	17.32	−1.00
c30728_g1	ZF-HD homeobox protein At4g24660-like (ZF-HD)	26.76	1.02	24.15	−0.03	21.80	−0.98
c33493_g1	ZF-HD homeobox protein At4g24660-like (ZF-HD)	71.60	0.80	69.79	0.32	64.39	−1.12
c35843_g1	ZF-HD homeobox protein At4g24660-like (ZF-HD)	13.56	1.15	10.95	−0.62	11.07	−0.54
c48188_g1	ZF-HD homeobox protein At4g24660-like (ZF-HD)	0.28	0.49	0.31	0.66	0.00	−1.15
c34004_g2	ZF-HD homeobox protein At5g65410-like (ZF-HD)	32.25	0.90	30.86	0.17	28.50	−1.07
c37708_g2	homeobox protein BEL1 homolog (BEL1)	6.16	−0.93	6.88	1.06	6.45	−0.13
c37708_g3	homeobox protein BEL1 homolog (BEL1)	4.56	−1.10	5.97	0.25	6.60	0.85
c4775_g1	homeobox protein, partial (HD)	4.23	1.12	3.93	−0.82	4.01	−0.30
**Autonomous**							
c32922_g1	flowering time control protein FY-like (FY)	20.08	0.99	19.44	0.01	18.78	−1.01
c38107_g2	flowering time control protein FPA-like (FPA)	8.25	1.15	0.98	−0.60	1.18	−0.55
c28761_g1	flowering time control protein FCA-like (FCA)	26.84	0.66	25.4	−1.15	26.71	0.49
c38768_g2	flowering time control protein FCA-like (FCA)	17.26	0.92	16.89	0.15	16.3	−1.07
c38623_g3	DRM1 protein (DRM1)	116.20	1.13	39.80	−0.76	55.01	−0.38
c34206_g2	protein FRIGIDA-like (FRI)	132.72	1.14	125.67	−0.44	124.46	−0.71
c35037_g1	protein FRIGIDA-like (FRI)	5.36	−1.08	5.78	0.19	6.01	0.89
c37853_g1	protein FRIGIDA-like (FRI)	6.2	0.93	5.75	−1.06	6.02	0.13
c19328_g1	FRIGIDA-like protein 4a-like (FRI4)	0	−0.58	0.76	1.15	0	−0.58
c39310_g1	FRIGIDA-like protein 5 (FRI5)	5.16	0.70	4.98	0.45	3.85	−1.15
**Photoperiod**							
c30945_g4	E3 ubiquitin-protein ligase COP1-like (COP1)	3.79	−1.02	7.31	0.98	5.67	0.05
c36345_g1	E3 ubiquitin-protein ligase COP1-like (COP1)	13.74	−1.13	17.6	0.35	18.71	0.78
c38384_g2	photoregulatory zinc-finger protein COP1 (COP1)	20.91	−0.44	26.21	1.14	20	−0.71
c31085_g1	circadian clock-associated FKF1 (FKF1)	10.67	−0.17	5.32	−0.90	19.71	1.07
c39712_g1	cryptochrome DASH, chloroplastic/mitochondrial-like, partial (CRY)	32.37	−1.00	39.68	1.00	36.06	0.01
c39765_g1	cryptochrome 2 (CRY2)	52.18	−0.81	55.53	1.12	53.05	−0.31
c40509_g3	zinc finger protein CONSTANS-LIKE 4-like (COL4)	117.03	0.38	118.27	0.76	112.04	−1.13
c18767_g1	zinc finger protein CONSTANS-LIKE 7-like (COL7)	1.61	−0.07	2.60	1.04	0.82	−0.96
c4034_g1	putative zinc finger protein CONSTANS-LIKE 11-like (COL11)	0.25	1.06	0.10	−0.13	0.00	−0.93
c11629_g1	zinc finger protein CONSTANS-LIKE 12-like (COL12)	0.40	−1.13	0.68	0.77	0.62	0.36
c34470_g3	zinc finger protein CONSTANS-LIKE 13-like (COL13)	26.31	−1.15	31.88	0.45	32.77	0.70
c35774_g1	zinc finger protein CONSTANS-LIKE 14-like (COL14)	47.93	−0.97	49.21	1.03	48.51	−0.06
c29880_g1	zinc finger protein CONSTANS-LIKE 16-like (COL16)	11.42	−0.93	14.22	1.06	12.54	−0.13
c30323_g1	zinc finger protein CONSTANS-LIKE 16-like (COL16)	3.54	−1.14	8.05	0.71	7.37	0.43
c34644_g1	transcription factor PIF1-like (PIF1)	6.38	0.21	6.70	0.88	5.76	−1.09
c39619_g1	transcription factor PIF1-like (PIF1)	5.25	−0.12	5.73	1.06	4.92	−0.93
c40293_g1	transcription factor PIF3 (PIF3)	21.97	−1.06	27.88	0.93	25.49	0.13
c38047_g1	transcription factor PIF5-like (PIF5)	19.33	0.97	16.69	0.05	13.59	−1.03
c19973_g1	protein PHYTOCHROME KINASE SUBSTRATE 1-like (PKS1)	2.85	0.01	4.44	1.00	1.23	−1.00
c40691_g1	chalcone synthase protein (CHS)	98.55	−0.82	128.96	−0.29	208.33	1.11
c39213_g1	EID1-like F-box protein 3-like (EID1)	34.15	0.34	23.20	−1.13	37.43	0.78
c23827_g1	mitochondrial import inner membrane translocase subunit TIM17-2-like (TIM17-2)	74.33	1.04	65.12	−0.95	69.09	−0.09
c35640_g1	cationic amino acid transporter 4 (CAT4)	10.17	−1.14	10.94	0.42	11.09	0.72
c31718_g1	cationic amino acid transporter 8, vacuolar (CAT8)	9.47	−0.79	9.79	−0.33	10.81	1.12
c39905_g2	cationic amino acid transporter 9, chloroplastic-like (CAT9)	14.25	−1.15	18.12	0.53	18.35	0.63
**Circadian rhythum**							
c26731_g1	early light-induced protein, chloroplastic-like (ELIP)	494.21	−0.53	825.57	1.15	475.1	−0.63
c28089_g1	early light-induced protein, chloroplastic-like (ELIP)	57.87	−0.35	97.05	1.13	46.77	−0.77
c37603_g1	protein CCA1-like (CCA1)	27.86	−0.28	68.82	1.11	11.65	−0.83
c63100_g1	late elongated hypocotyl-like (LHY)	0.24	1.15	0.00	−0.58	0.00	−0.58
c39324_g1	protein GIGANTEA-like (GI)	97.02	0.25	65.89	−1.10	110.67	0.85
c38696_g1	protein EARLY FLOWERING 3-like (ELF3)	28.76	−1.09	32.17	0.22	33.84	0.87
c33120_g1	bZIP transcription factor 60 (bZIP60)	92.57	1.07	55.32	−0.92	69.8	−0.15
c25903_g1	protein STRUBBELIG-RECEPTOR FAMILY 6-like (SRF6)	78.56	1.15	63.48	−0.59	63.76	−0.56
**Temperature**							
c32035_g1	B3 domain-containing transcription factor VRN1-like (VRN1)	5.41	1.15	3.02	−0.56	2.98	−0.59
c32197_g1	B3 domain-containing transcription factor VRN1-like (VRN1)	10.48	1.00	8.4	0.00	6.29	−1.00
c23355_g2	B3 domain-containing transcription factor VRN1-like (VRN1)	1.59	1.12	0.54	−0.79	0.79	−0.33
c27896_g1	B3 domain-containing transcription factor VRN1-like (VRN1)	38.48	1.15	33.62	−0.68	34.17	−0.47
c31063_g2	protein VERNALIZATION INSENSITIVE 3-like (VIN3)	16.63	−1.06	18.63	0.13	19.96	0.93
c39974_g4	protein VERNALIZATION INSENSITIVE 3-like (VIN3)	28.65	1.05	25.73	−0.94	26.96	−0.10
c19924_g1	Temperature-induced lipocalin (TIL)	0.15	−0.89	3.92	1.08	1.5	−0.19
c40025_g1	heat shock 70 kDa protein 15-like (HSP70)	169.05	−0.37	204.83	1.13	159.88	−0.76
c65251_g1	70 kDa heat shock protein (HSP70)	0.11	0.24	0.16	0.86	0.00	−1.10
c27640_g1	heat shock protein 83-like (HSP83)	205.98	0.93	134.65	0.12	30.81	−1.06
c27956_g1	heat stress transcription factor A-6b-like isoform 1 (HSF)	12.30	0.20	12.93	0.88	11.10	−1.09
c26273_g1	cytosolic class II small heat-shock protein HSP17.5 (SHSP)	349.85	1.03	198.62	−0.06	72.70	−0.97
c29035_g1	histone H2A-like (H2A)	567.29	1.00	487.09	−1.00	527.75	0.01
c40510_g1	HEAT repeat-containing protein 5B-like (HEATP5)	15.51	0.15	16.24	0.92	14.37	−1.06
